# ECM-Regulator *timp* Is Required for Stem Cell Niche Organization and Cyst Production in the *Drosophila* Ovary

**DOI:** 10.1371/journal.pgen.1005763

**Published:** 2016-01-25

**Authors:** John R. Pearson, Federico Zurita, Laura Tomás-Gallardo, Alfonsa Díaz-Torres, María del Carmen Díaz de la Loza, Kristian Franze, María D. Martín-Bermudo, Acaimo González-Reyes

**Affiliations:** 1 Centro Andaluz de Biología del Desarrollo, CSIC/Universidad Pablo de Olavide/JA, Sevilla, Spain; 2 Departamento de Genética e Instituto de Biotecnología, Universidad de Granada, Centro de Investigación Biomédica, Granada, Spain; 3 Department of Physiology, Development and Neuroscience, University of Cambridge, Cambridge, United Kingdom; New York University, UNITED STATES

## Abstract

The extracellular matrix (ECM) is a pivotal component adult tissues and of many tissue-specific stem cell niches. It provides structural support and regulates niche signaling during tissue maintenance and regeneration. In many tissues, ECM remodeling depends on the regulation of MMP (matrix metalloproteinase) activity by inhibitory TIMP (tissue inhibitors of metalloproteinases) proteins. Here, we report that the only *Drosophila timp* gene is required for maintaining the normal organization and function of the germline stem cell niche in adult females. *timp* mutant ovaries show reduced levels of both *Drosophila* Collagen IV α chains. In addition, tissue stiffness and the cellular organization of the ovarian niche are affected in *timp* mutants. Finally, loss of *timp* impairs the ability of the germline stem cell niche to generate new cysts. Our results demonstrating a crucial role for *timp* in tissue organization and gamete production thus provide a link between the regulation of ECM metabolism and tissue homeostasis.

## Author Summary

The extracellular matrix (ECM) offers signals and support to stem cell niches, local microenvironments that provide these cells with necessary factors for their survival. The ECM also helps shaping and maintaining tissues and organs in adult animals. Because the repair of damaged tissue or the replenishment of cell lineages in functional organs requires significant cellular rearrangements, ECM remodeling has to be tightly coordinated with stem cell niche activity. By studying Timp, a regulator of ECM remodeling, we have discovered that the *Drosophila timp* gene is required to maintain ECM composition and biophysical properties and the organization of the female germline stem cell niche. Because loss of *timp* prevents proper gamete production in experimental ovaries, our results thus link ECM metabolism and tissue homeostasis.

## Introduction

The extracellular matrix (ECM) is an essential component of adult stem cell niches, and hence a fundamental player in tissue homeostasis, as it regulates stem cell fate by mediating signal delivery and by providing matrix-directed differentiation [1, 2]. In addition, the ECM is also required for tissue structure and integrity, organ morphogenesis and signaling [3]. Thus, we might expect ECM degradation and remodeling to be tightly coordinated during organogenesis and tissue maintenance.

Matrix metalloproteinases (MMPs) are a class of well-known proteolytic enzymes that are able to degrade most ECM components and promote ECM turnover [4, 5]. Because of their functions in ECM remodeling, MMPs play key roles in development and regeneration, as shown for branching morphogenesis, angiogenesis and wound healing [6]. Synthesized as inactive zymogens, MMPs are normally activated extracellularly and are classified depending on their substrate specificity or on the presence of structural motifs. Thus, MMPs can degrade fibrillar collagen (collagenases) or denatured collagen (gelatinases), they can process non-collagen components of the ECM such as fibronectin and a number of membrane-bound MMPs have also been described. MMPs are typically regulated by the Tissue inhibitor of metalloproteinases (TIMP) family proteins, which are secreted multifunctional proteins that engage MMPs non-covalently to block access to their catalytic domain. Given the importance of the correct control of MMP activities, it has been proposed that an imbalance between TIMP and MMP molecules may lead to pathological conditions [7–9]. Furthermore, the control of stem cell proliferation by MMP activity has been recently reported in the ovarian niche in *Drosophila*, underscoring the importance of ECM metabolism in stem cell niche regulation [10].

While vertebrates contain twenty-six MMPs and four TIMPs, the *Drosophila melanogaster* genome possesses two MMPs (*Mmp1* and *Mmp2*) and a single *timp* gene [11]. Loss of *timp* function dramatically affects vitality and fertility of *Drosophila* adults, as mutant flies show a much-reduced lifespan and a ten-fold diminution in egg deposition [12]. *timp* mutant adults also display morphological defects, visible in the presence of autolyzed tissue in the abdominal cavity and in their inflated wings, a phenotype consistent with a role for Timp in ECM integrity and remodeling.

**Fig 1:**
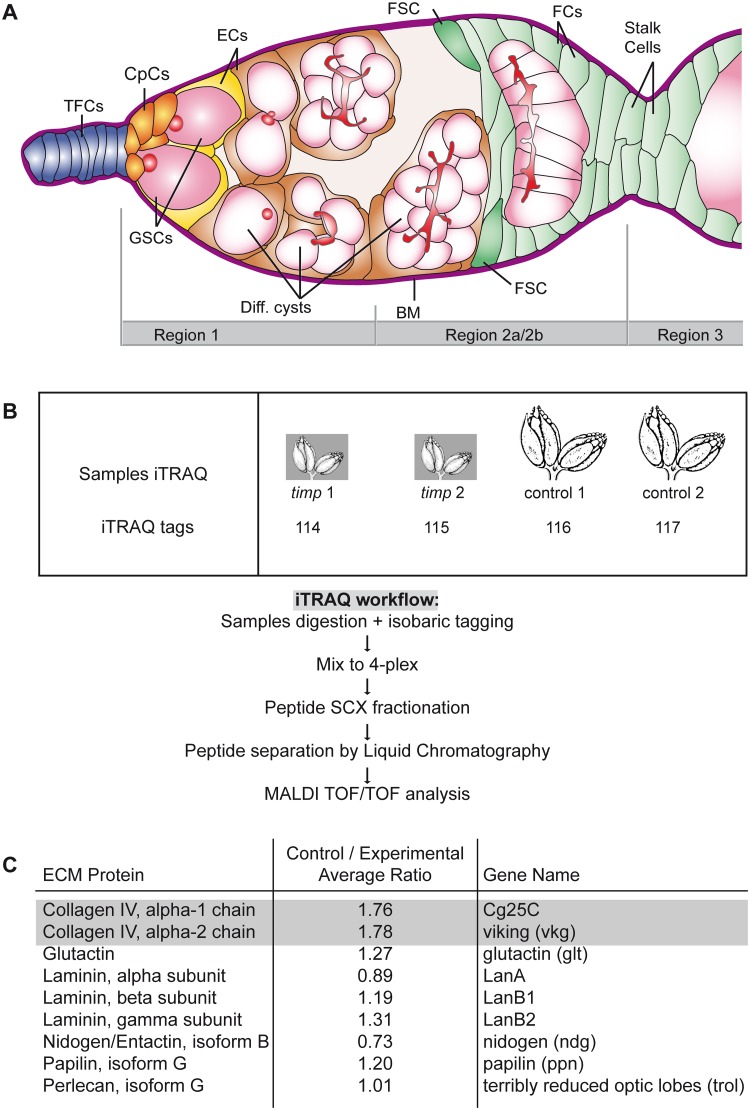
Experimental design of the iTRAQ 4-plex labeling. (A) Diagram of a wild-type germarium. The arrangement of somatic and germline-derived cell types of the germarium and the three regions in which germaria are subdivided are shown. Region 2 is further subdivided into 2a and 2b, depending on the shape of the 16-cell cysts (in 2b they stretch across the width of the germarium). (B) iTRAQ experiment workflow. (C) Table of the ECM components identified in the iTRAQ analysis. Only the two Collagen IV α chains are significantly underrepresented in the experimental tissue. TFCs: terminal filament cells; CpCs: cap cells; ECs: escort cells; FSC: follicle stem cell; FCs: follicle cells; GSCs: germline stem cells; Diff. cysts: differentiating cystoblasts, 4- and 16-cell cysts; BM: basement membrane. See also S1 and S2 Figs, S1 and S2 Tables.

The *Drosophila* female grows two ovaries in its abdomen. Each ovary consists of a series of egg-producing tubules termed ovarioles in which newly generated egg chambers or follicles develop. Each ovariole contains a specialized structure at the anterior apex—the germarium—home to germline and follicle stem cells. Egg chambers are assembled throughout the female’s life span in the germarium (Fig 1A). Developing egg chambers are composed of 16-cell germline cysts enveloped by a monolayer of somatic cells that form the follicular epithelium. Adjacent egg chambers are connected by a string of 5–8 somatic cells organized in a one-cell wide filament known as the interfollicular stalk. The germarium and the concatenated egg chambers are surrounded by a basement membrane, a specialized ECM that offers structural support and constitutes a substrate for tissue migration in the ovary [13, 14]. Basement membranes are rich in type IV Collagen, Laminins, Perlecan, Nidogen, and they may contain other extracellular matrix components such as Papilin, BM-40 or Glutactin [15, 16]. In addition to the basement membrane, the germarium also contains a significant amount of interstitial matrix, accumulated between the different cell types that reside within it. Because both the interstitial matrix and the basement membrane are continuously remodeled during normal oogenesis to allow egg chamber formation and maturation [14] (our unpublished observations), the *timp* gene and the effector proteins Mmp1 and Mmp2 are likely to play a role in female gametogenesis in *Drosophila*.

The germarium constitutes a *bona fide* niche for germline and somatic stem cells [17, 18]. At the anterior tip of the structure, a limited number of germline stem cells (GSCs) are kept within the area of influence of the stromal components of the niche, the terminal filament cells, the cap cells and the escort cells (Fig 1A) [19, 20]. Several signaling pathways that originate in the cap cells and escort cells eventually act on the receiving germline to regulate the maintenance of the GSC population within the niche, controlling their proliferation and daughter cell differentiation. In spite of the myriad of published studies focusing on the signals in the germarium, so far we lack an understanding of the roles that the ECM and its metabolism play in the correct functioning of the ovarian niche. Furthermore, considering the fact that the ECM is a key component of vertebrate stem cell niches and that the physical properties of the extracellular niche influence cell fate, a detailed analysis of ECM remodeling in a stem cell niche is bound to have wide implications in more complex systems [2, 21]. Here, we have used a multidisciplinary approach to examine the role of *timp* in the *Drosophila* ovary. Considering the canonical function of *timp* in ECM remodeling, our results demonstrating that *timp* is necessary for tissue organization and gamete production provide a link between regulation of ECM metabolism and proper tissue homeostasis *in vivo*.

## Results

### A proteomic approach identifies *timp* as a regulator of Collagen IV levels

Given the strong evidence linking Timp to the metabolism of ECM components, we set out to identify ECM proteins that are present in different amounts in control and *timp* mutant ovaries and to quantitate their relative levels. We performed in duplicate a 4-plex iTRAQ (isobaric Tags for Relative and Absolute Quantitation) analysis of our samples (Fig 1B). Since null *timp* 1-week old adult ovaries were significantly smaller than sibling controls we decided to use the *ovo*^*D1*^ dominant mutation, which blocks oogenesis at stage 4–5 of development [22], and thus compare gonads of similar size and containing developing egg chambers of roughly equivalent developmental stages. We used *ovo*^*D1*^/+;; *timp*^*28*^/TM3 ovaries as control samples and *ovo*^*D1*^/+;; *timp*^*28*^/Df as experimental tissue (S1A Fig). Utilizing the MASCOT search engine, we identified 644 proteins, which allowed the quantitation of protein products corresponding to 359 genes. We recognized 10 proteins with control/experimental average expression ratios ≤0.50 and 38 with ratios ≥1.50, corresponding to a total of 47 genes (S1 Table). Thus, 13.4% of the identified genes were either under- (2.8%) or over-represented (10.6%) in control *versus timp*-null ovaries. A bioinformatic search for significant functional pathways and the association of enriched biological annotations to the gene list using PANTHER and GeneCodis tools failed to identify any statistically overrepresented functional pathways or protein networks in the iTRAQ data set. However, the Gene Ontology analysis of the selected candidates indicated several facts. First, three genes with putative proteolytic activity (CG5618, *psa* and *DppIII*) were over-represented in *timp* mutant ovaries. Interestingly, transmission electron-microscopy studies of mutant ovaries showed frequent cellular degeneration not observed in control samples. Escort cells and follicle cells often displayed clear cytoplasms, multi-lamellar bodies and multi-vesicular vacuoles containing cell debris (S4M and S4N Fig). Second and most relevant to the aim of this work, proteins corresponding to nine ECM components were identified by the iTRAQ experiments. Of those, only the two collagen IV α chain homologues present in *Drosophila*, Cg25C (α1) and Viking (α2) [23, 24] were significantly changed, as they were specifically reduced in experimental tissues (Fig 1C).

In order to confirm that the proteins identified in the iTRAQ study represented changes due to the loss of *timp* activity, we performed a series of tests. First, we used an LTQ-Orbitrap ion trap mass spectrometer to compare a fraction of the proteome of 1-week old *w*^*1118*^ (wild-type) ovaries with that of *ovo*^*D1*^/+;; *timp*^*28*^/TM3 of the same age, as determined by the iTRAQ study. The LTQ-Orbitrap analysis identified 610 proteins with a MASCOT score above 50 and a peptide hit ≥ 2.380 of these (62.3%) were also found in the *ovo*^*D1*^/+;; *timp*^*28*^/TM3 iTRAQ analysis (S1B–S1D Fig and S1 Table). A Gene Ontology analysis utilizing the GeneCodis tool to search for biological annotations significantly associated to both sets of identified genes rendered similar results, with over 90% of the clustered protein hits in the iTRAQ and LTQ approaches falling in the same Biological Process categories (S1C and S1D Fig). These results corroborated that the iTRAQ experiment identified a representative proteome of the normal tissue. Second, we performed two-dimensional DIfference Gel Electrophoresis (2D-DIGE) to validate proteins differentially expressed either in control ovaries (*w*^*1118*^;; *timp*^*28*^/TM3) or in experimental ones (*w*^*1118*^;; *timp*^*28*^/Df). Protein samples isolated from both types of ovaries were labeled with different fluorescent dyes and separated in two-dimensional electrophoresis, which allowed the isolation and identification by mass-spectrometry of three proteins. Two of these were up-regulated in control samples (Chorion protein S16 and Vitelline membrane 26Aa, with standardized logarithm abundance ratios of 3.02 and 3.67, respectively) and the third one was more abundant in *timp* mutant ovaries (Regucalcin, average ratio of -1.56). In agreement with these findings, the iTRAQ experiment identified another of the chorion proteins in the *Drosophila* genome (S15) as over-represented in control ovaries, while Regucalcin was found over-expressed in mutant ovaries (S2A–S2C Fig and S2 Table). Third, to rule out the possibility that the genetic combination used in the iTRAQ study (a deletion for the *timp* gene in trans to a larger deficiency for the locus) could identify as false-positives flanking genes removed by the deficiency or the deletion, we checked individually all the genes removed by both deletion and deficiency (*Syn* and *timp*) or by the deficiency alone (CG12814, CG12817, CG12420, CG34107, CG42795, CG3999, CG43143, CG6293, CG12818, CG12592, CG18545, CG31406, *Best1*, *Sirt6*, *pont*, *Bruce*, *sle* and *jumu)*. None of the proteins encoded by these genes were identified in the iTRAQ study.

Altogether, the above experiments link the absence of the *timp* gene and the selective loss of ECM Collagen IV from mutant ovaries, hence suggesting a substantial connection between collagen IV metabolism and Timp activity.

### Zymography assays confirm *timp* as a regulator of Collagen IV proteolysis

**Fig 2:**
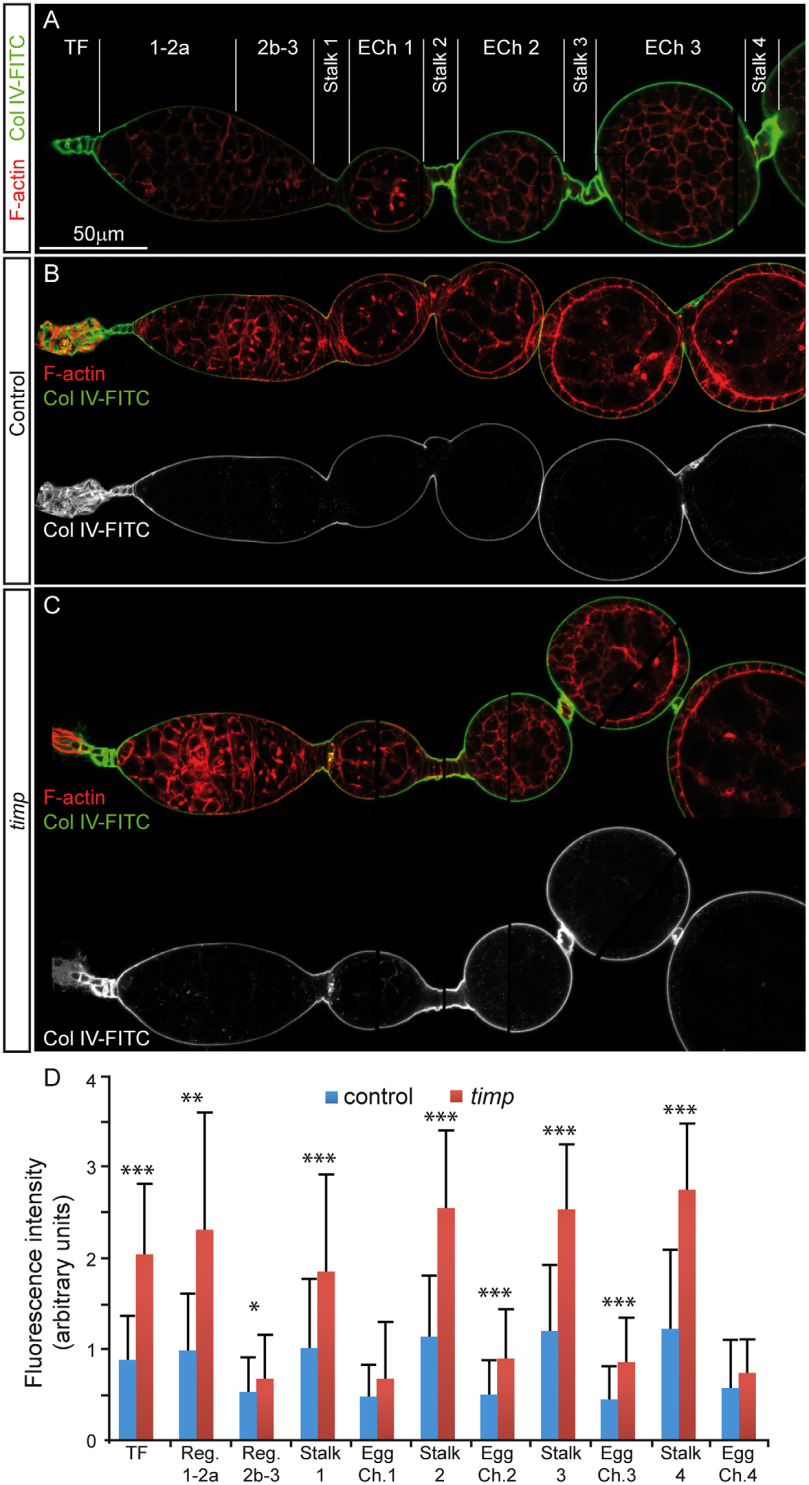
*In vivo* zymography reveals increased collagenase activity in *timp* mutant ovaries. Control and *timp* mutant ovaries were incubated in culture medium with human Collagen IV-FITC for two hours then fixed and stained to show filamentous actin (F-actin). Green fluorescence corresponds to Col IV-FITC molecules cleaved by zymogen activity in the ovaries. (A) Fluorescence along each ovariole was measured at multiple positions within the following regions: the TF (terminal filament, 12 measurements/ovariole), germarial regions 1-2a (15 measurements/ovariole), and 2b-3 (9 measurements/ovariole), interfollicular stalks (12 measurements/ovariole) and successive egg chamber s (ECh, 12 measurements/ovariole). 5 control and 3 experimental ovarioles were measured. (B) Control ovariole. Note the Col IV-FITC staining decorating the basement membrane and the slightly increased signal in the stalks. (C) *timp* mutant ovariole. Note that the Col IV-FITC staining, particularly in the anterior tip of the germarium and in the stalks, is stronger than the control. (D) Graph showing the average fluorescence intensity in arbitrary units along the anterior-posterior axis of the ovariole. Images are composites of several focal planes. P-values were obtained using a Student’s *t*-test. P values <0.05 were considered statistically significant (*:P<0.05, **:P<0.005, ***:P<0.0005). Unless otherwise stated, the genotypes used in this and the remaining figures are: control *w*^*1118*^;; *timp*^*28*^/TM3 and *timp* mutant *w*^*1118*^;; *timp*^*28*^/Df(3R) ED5472. See also S2 and S3 Figs.

To test if *timp* is involved in the regulation of Collagen IV turnover, most likely by preventing excessive MMP-mediated degradation, we performed an *in vivo* zymography assay on live ovaries. We incubated 1-week old control and *timp* mutant ovaries with human Collagen IV-FITC, fixed them, co-stained them with Rhodamine-Phalloidin and measured the intensity of the FITC signal accumulated in the basement membrane of the treated ovaries. FITC fluorescence levels are proportional to Collagen IV-FITC cleavage and the proteolytic activity of the target tissue. Treatment of control and mutant ovaries in parallel rendered significantly different fluorescent intensity values, with the ECM of *timp* ovaries consistently showing stronger signal levels (Fig 2). Importantly, pre-incubating the samples 30’ with 1mM 1, 10-Phenanthroline—a general MMP inhibitor (see for instance [25]—blocked Collagen IV-FITC degradation (S3 Fig). Thus, assuming that Collagen IV-FITC incorporates at equal levels in both control and experimental BMs, we conclude that *Drosophila timp* modulates Collagen IV degradation by regulating MMP activity in the ovarian ECM.

### ECM polarization in older follicles seems unaffected in *timp* mutants

The germarium is subdivided into 3 regions: Region 1 contains the GSCs and proliferating germ line; Region 2 is populated by 16-cell germline cysts that eventually become enveloped by follicle cells, the progeny of the two follicle stem cells located in this Region; in Region 3 cysts bud off to form developing egg chambers (Fig 1A). To determine if the distribution of major ECM components in anterior ovarian tissues requires *timp* activity, we studied the distribution of basement membrane components in control germaria by immunostaining. We found strong accumulation of Collagen IV α2 (Vkg), Perlecan, Laminin β-chain and Laminin γ-chain surrounding the terminal filament and GSC niche region. Weaker staining was generally observed along the exterior of Regions 1 and 2, with stronger levels accumulating on the basal side of the nascent follicular epithelia in Region 3 and surrounding the interfollicular stalks. The distribution of the above ECM proteins in *timp* null mutant females, even as old as 3–4 weeks and exhibiting severe morphological defects, was generally undistinguishable from controls (S4 and S5 Figs). Transmission Electron Microscopy (TEM) analysis on 3–4 week old control and mutant germaria showed an equal accumulation of electron dense material consistent with that observed by confocal microscopy for Collagen IV, Perlecan and Laminins in the GSC niche area and in the lateral regions (S6 Fig).

During mid-oogenesis, the basement membrane of the follicular epithelium serves as a substrate for follicle rotation, a global migration process by which egg chambers execute three complete turns while transitioning from a spherical shape to an elongated egg [14]. During rotation, follicle cells basally secrete matrix components that assemble at the basement membrane, creating a polarized extracellular network perpendicular to the long axis of the developing egg. This “molecular corset” acts to restrict central region growth of the egg chamber while allowing expansion towards the poles [26]. Since *timp* is required for Collagen IV metabolism, a major component of the basement membrane, we tested whether Collagen IV secreted by the follicle cells could assemble properly at the basement membrane in the absence of *timp* activity. To this end, we first checked that the polarized accumulation of Collagen IV and Perlecan in rotating egg chambers from 2-week old mutant females was indistinguishable from controls (S4C, S4D, S4J and S4K Fig), suggesting again the absence of gross malformations in ECM organization, at least as judged by confocal microscopy. Second, we performed FRAP (Fluorescent Recovery After Photobleaching) experiments utilizing a GFP protein trap in the *viking* gene to monitor the deposition of Collagen IV. We measured fluorescence recovery at three different time points, from stages 1–3, when most egg chambers have not yet initiated rotation ([14] and our unpublished observations, but see [27]) and during rotation (stages 5–6 and 7–8). Before stage 5, neither control nor mutant egg chambers showed any sign of recovery in the bleached regions 100 min. after photobleaching. In contrast, stage 5–8 control and *timp* mutant follicles displayed detectable deposition of Collagen IV:GFP in the photobleached area 100 min. after bleaching (S7 Fig). Since we could not detect statistically significant differences between control and *timp* mutant samples 100 min. after bleaching, we conclude that the general architecture and organization of the ECM during oogenesis is not grossly affected by the absence of *timp* function.

### Ectopic *timp* expression results in elongated germaria and egg chamber fusions

**Fig 3:**
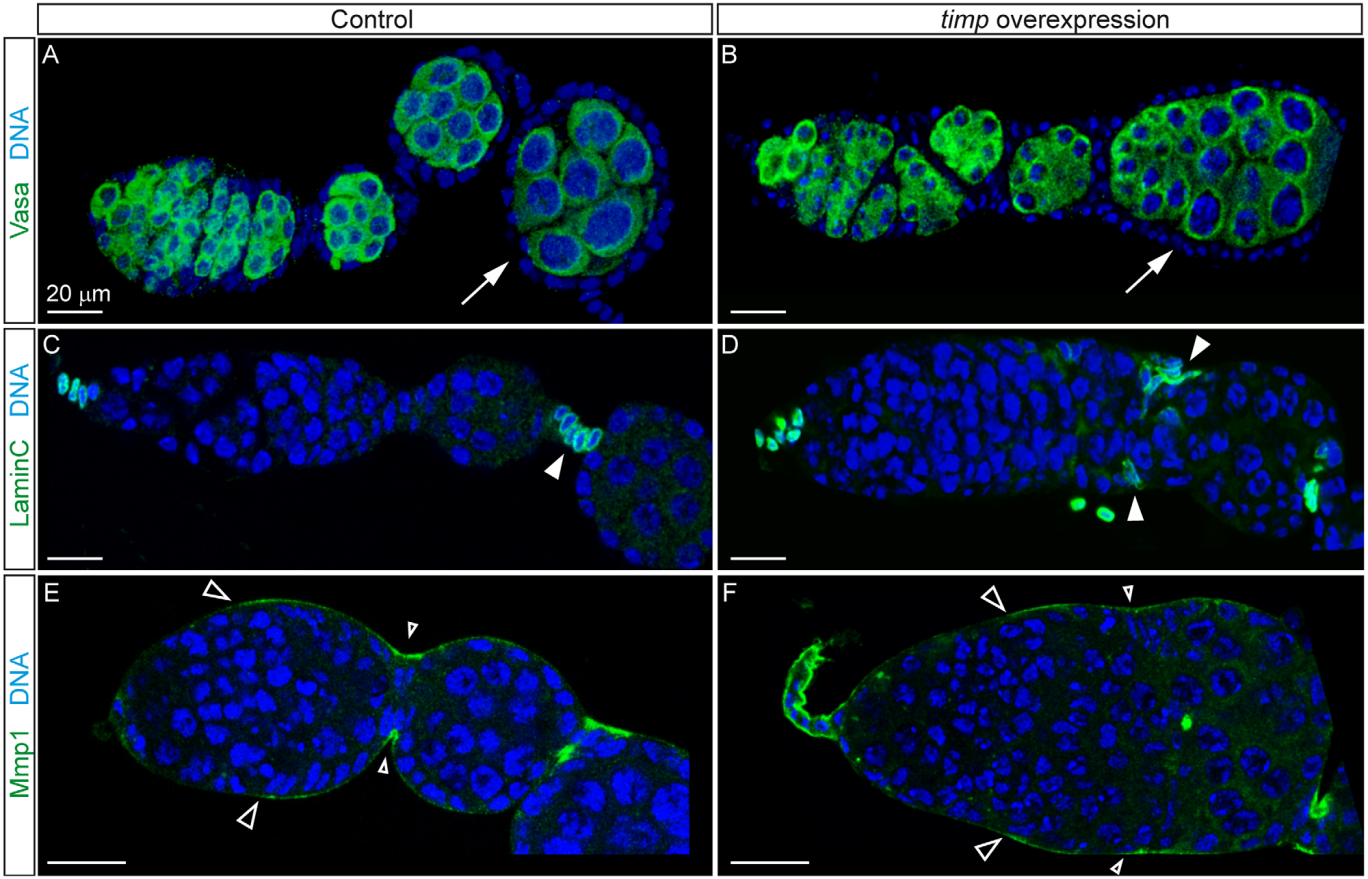
*timp* over-expression impairs cyst encapsulation without affecting cell fate acquisition. (A, B) Ovarioles from control (no heat-shock) and experimental (daily heat-shock regime for 2 weeks post-eclosion) animals carrying *UAS-timp* and *hs-Gal4*. Arrows compare a normal egg chamber containing 16 germ cells with an abnormal, fused egg chamber encasing 32 germ cells. (C, D) Control (*c587-Gal4*) and experimental (*c587-Gal4; UAS-timp*) ovarioles grown at 18°C and then switched to 25°C for 9 days. Arrowheads indicate Lamin C-positive stalk cells. (E, F) Control (*c587-Gal4*) and experimental (*c587-Gal4; UAS-timp*) ovarioles grown at 18°C and aged 4 weeks at 25°C. Large, empty arrowheads show Mmp-1 accumulation from region 2. The ring of strong Mmp1 staining that coincides with the region contracting to pinch off a new egg chamber in controls is absent in *timp*-overexpressing germaria (small, empty arrowheads). Occasionally, the TF of experimental germaria show a prominent accumulation of Mmp1 staining, indicating that *timp* overexpression may affect Mmp1 localization in this region. Images can be composites of several focal planes.

The absence of gross alterations in the localization of ECM proteins in mutant germaria could suggest that changes to MMP activity resulting from the lack of *timp* are highly localized (e.g. affecting only specific cell types) and/or modify ECM properties without affecting its overall distribution. Overexpression of *timp* in the somatic component of otherwise wild-type ovaries using a *UASt*-*timp* insertion [28] combined with either a *heat-shock Gal4* or the germarium-specific Gal4 *c587* [29] resulted in the formation of compound follicles containing more than one germline cyst (Fig 3A and 3B). This fusion phenotype could be caused by a defect in the encapsulation of the cysts in germarial Region 2 or by a failure in the specification of stalk cells that separate adjacent egg chambers [30]. Significantly, *c587-Gal4* expression, which produced the strongest *UASt-timp* induced phenotypes, is specific to a subset of somatic cells in the germarium [29], implying the need for a fine regulation of extracellular proteolytic activity in anterior ovarian tissues during early cyst formation. Examination of ovaries containing *timp*-induced egg chamber fusions revealed the presence of cells expressing the stalk cell marker Lamin C towards the periphery of the chambers, in the approximate position where stalk cells would normally be located (Fig 3C and 3D). These observations emphasize the importance of the fine-regulation of *timp* function in the somatic cells of the germarium for the correct organization of stalk cells and for the encapsulation of new cysts, a process that requires extensive cell migration and ECM remodeling. Importantly, enlarged germaria induced by *timp* overexpression lack the strong accumulation of Mmp1 in the region where the first interfollicular stalk would form (Fig 3E and 3F; see also Fig 7 for a detailed description of Mmp1 localization), supporting the idea that *timp* overexpression may affect Mmp1 activity during cyst encapsulation.

### Tissue stiffness in the germarium is determined by *timp* activity

**Fig 4:**
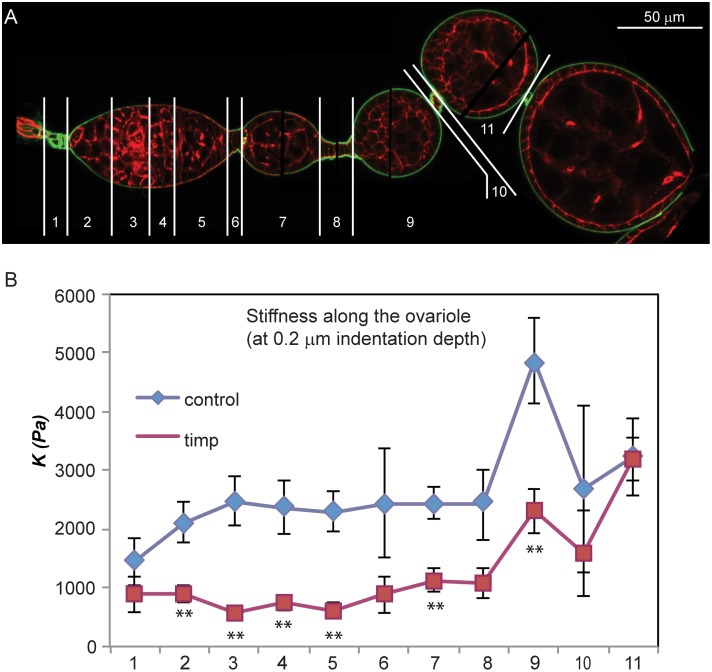
*timp* mutant ovarioles have altered physical properties. (A) Subdivision of regions for AFM-based analysis of ovariole tissue stiffness. (B) Comparison of the apparent elastic modulus *K* at discrete points along 3–4 week old wild-type (n = 7) and *timp* null mutant ovarioles (n = 12) *ex-vivo*. Mutant ovarioles exhibited significantly lower levels of tissue stiffness throughout the regions tested. Differences were most severe in the germarium, early egg chambers and their associated interfollicular stalks. Results shown refer to an indentation depth of 0.2 μm. The image is a composite of several focal planes. ** = *p* values of two-tailed t-tests <0.01. See also S6 Fig.

Our data suggest that the dynamics of Collagen IV protein accumulation are affected in the absence of the *timp* gene. As collagen is an important contributor to tissue stiffness [21], we assessed the mechanical properties of control (n = 7) and mutant (n = 12) ovarioles using Atomic Force Microscopy (AFM)-based indentation measurements. We found a significant reduction in the stiffness along mutant germaria, including the GSC niche and the area where the follicle stem cells reside, as well as in the follicular epithelium of early egg chambers and their interfollicular stalks (Fig 4 and S8 Fig). Our AFM data demonstrate that the absence of *timp* results in the alteration of physical properties in germarial ECM and early egg chambers. As reported below, the decrease in tissue stiffness may explain the striking morphological defects in tissular organization observed in mutant germaria and their impaired ability to generate new cysts.

### *timp* is necessary for oogenesis and to maintain ovarian niche tissue stability

**Fig 5:**
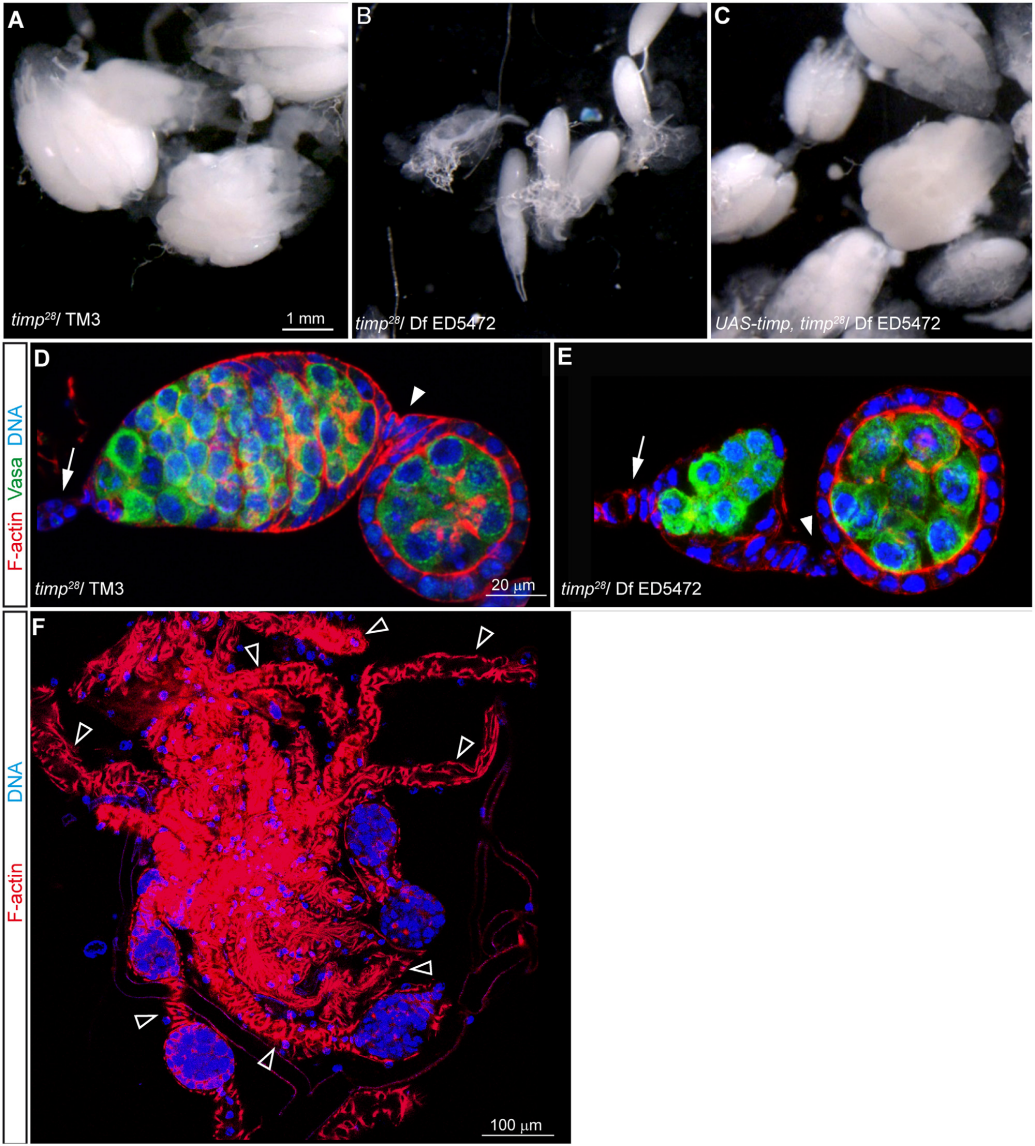
Ovaries from *timp* mutant females are smaller and lack integrity. (A) Ovaries dissected from 2-week old control females. (B) Small disorganized ovaries dissected from a 2-week old *timp* null mutant. (C) Normal ovary morphology restored in ovaries dissected from 2-week old females lacking the endogenous *timp* gene but carrying a *UASt-timp* transgene. (D) Germarium from a 2-week old control ovary stained with Rhodamine-Phalloidin to visualize F-actin (red), Vasa to label de germline cells (green) and Hoescht to mark DNA (blue). (E) Germarium from a 2-week old mutant female stained as before. Note the marked decrease in the number of developing cysts within the germarium. (F) Ovary from a 4-week old mutant female stained to visualize the outline of the tissue and DNA. Empty arrowheads point to depleted ovarioles containing very few or no follicles. Arrows: terminal filament. Arrowheads: first interfollicular stalk. Images can be composites of several focal planes.

**Fig 6:**
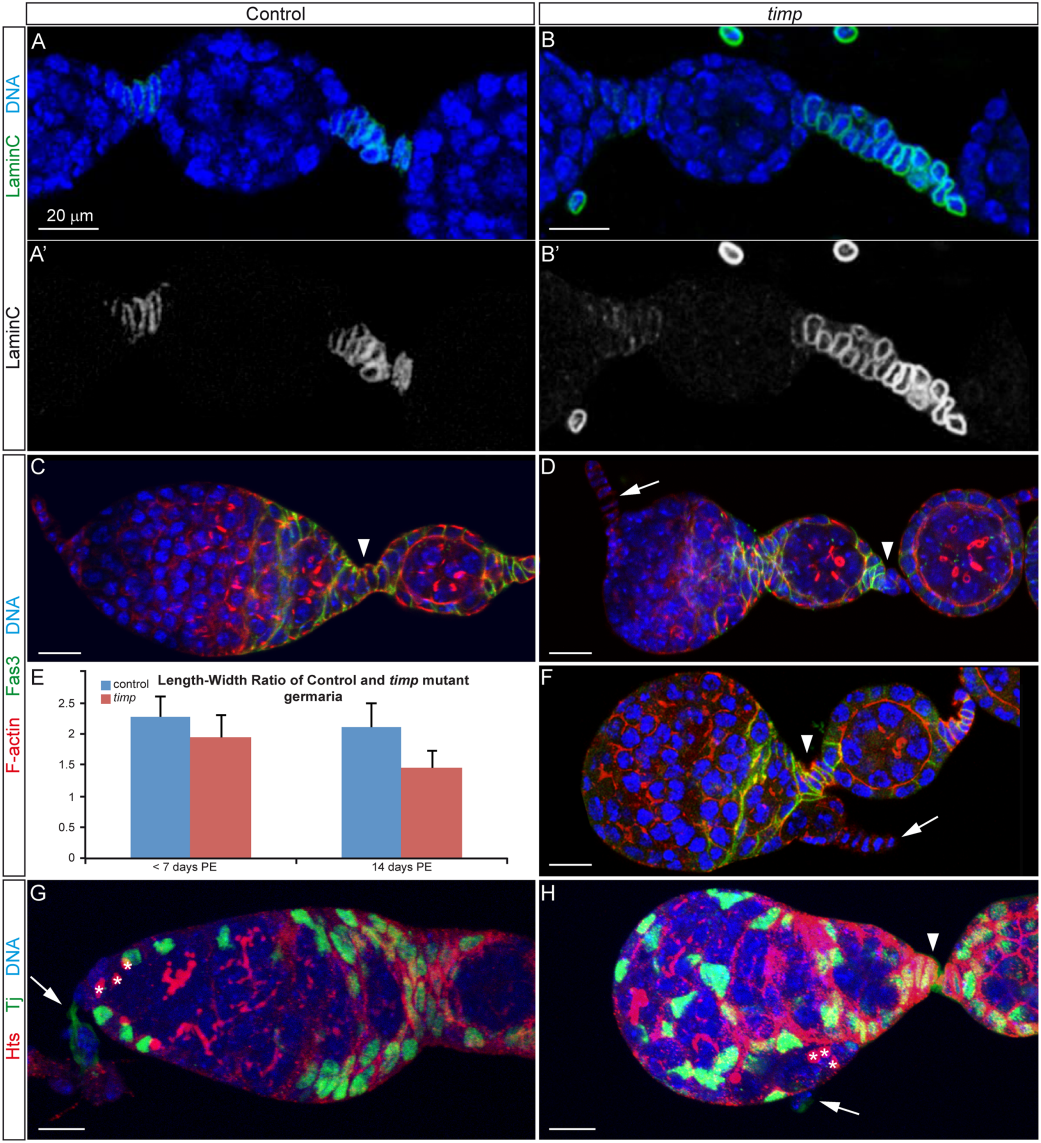
*timp* is required to maintain germarium organization and shape. Distribution of the stalk-cell marker Lamin C in control (A, A’) and *timp* mutant ovarioles (B, B’). (C) Normal germarium morphology in ovaries dissected from 2-week old control females. FasIII (green) is expressed by pre-follicle cells in the germarium. Phalloidin-labeled actin (red) and DNA (blue) are shown. (D) Abnormal rounded shape of germaria commonly seen in ovaries dissected from 2-week old mutant females. In most cases, despite the altered shape, overall organization of the terminal filament and FasIII expressing cells seem broadly similar to wild-type germaria. (E) Graph quantifying the changing shape of *timp* mutant germaria 1–7 and 14 days post-eclosion. Differences in length-to-width ratios between control and mutants are statistically significant (*p* value of two-tailed t-tests <0.001 for 14-day old germaria). (F) Abnormal organization of distinct domains of a *timp* mutant germarium. In extreme cases, the terminal filament is located to the posterior of region 3, adjacent to egg chambers already pinched off from the germarium. This highly unusual phenotype has not been described previously and is never observed in wild-type germaria. (G, H) The distribution of Escort Cells (labeled in green with the Tj protein) seems normal in aberrantly shaped germaria. The position of GSCs, as determined by their spectrosomes (anti-Hts staining, red) indicates that these cells are still associated to the terminal filament and cap cells, albeit in the example in (H) they are adjacent to follicle cells. Arrows: terminal filament. Arrowheads: first interfollicular stalk. Asterisks: GSC spectrosomes. Images can be composites of several focal planes. See also S2 Table.

Null *timp* mutant females are sub-viable, semi-sterile and grow smaller ovaries, a phenotype that can be rescued by the leaky expression from a *UAS-timp* transgene in absence of a Gal4 driver (Fig 5A–5C). Upon closer inspection of 4-week old mutant ovaries, we observed depleted ovarioles containing very few or no maturing follicles (Fig 5D–5F), suggesting that oogenesis is severely affected in the absence of the *timp* gene and that this depends on the age of the tissue. We thus decided to analyze in greater detail the effects of *timp* loss-of-function in aging ovaries. First, we focused on the development of interfollicular stalks. While in control ovaries they are formed by a one-cell wide filament of 5–8 cells, *timp* mutants frequently showed abnormally long interfollicular stalks containing 9 or more cells (Fig 6A and 6B). Lamin C, a marker for differentiated stalk cells, accumulated normally in these additional cells from *timp* null mutants, suggesting alterations in cell recruitment rather than a non-specific accumulation of cells in mutant stalks. The frequency of ovarioles containing large 9+ cell stalks augmented dramatically with time, increasing from ~15% to ~50% in ovaries dissected from 1 and 7 day old *timp* females, respectively (S2 Table).

Second, we analyzed niche organization in detail. Wild-type germaria typically exhibited an elongated shape with a length (anterior-posterior) to width ratio of approximately 2:1 (Fig 6C and 6E and S2 Table). In ovaries dissected from 1–7 day old *timp* null mutant females, germaria were essentially indistinguishable from controls. In contrast, ovaries from older mutants showed significant alterations to germarium morphology: 59.1% of germaria from 2-week old mutants displayed a much more rounded appearance with a length-width ratio of 1.45:1 (Fig 6D–6F and S2 Table). In such rounded germaria, many gross abnormalities in stem cell niche organization were observed. While control germaria always showed a stereotyped arrangement of cell types, with the terminal filament and cap cells at the tip (Fig 6C), we found mutant germaria where these cells were abnormally positioned. The example in Fig 6F depicts a germarium in which the terminal filament and cap cells are adjacent to an interfollicular stalk and to a differentiated egg chamber. Because the positioning of the GSCs adjacent to the cap cells at the base of the terminal filament is maintained in mutant germaria, the abnormal arrangement observed in a large proportion of aged *timp* germaria implied that GSCs were positioned in close proximity to follicle cells (Fig 6G and 6H and S2 Table), while the intercalation of Escort Cells in between germline cysts seemed normal. The changes in shape and the alterations in its organization in older mutant adults show that *timp* plays a role in maintaining germarium structure.

### *timp* is required for proper ovarian niche activity

To analyze the outcome of niche activity, we determined whether loss of *timp* affected the ability of mutant ovaries to produce new germline cysts. We counted the number of 2-, 4-, 8- and 16-cell cysts present in 10-day and 3-week old germaria. Compared to controls, *timp* mutants showed a clear diminution in the number of cysts populating 3-week old germaria (controls 9.5±2.2 cysts/germarium, n = 19; mutants 5.8±3.5 cysts/germarium, n = 29. S3 Table). This difference is not a consequence of a drop in the number of GSCs present in the niche, as control and *timp* hemizygous germaria did not show statistically significant differences in GSC numbers either 10 days or three weeks after eclosion (S3 Table). This result indicates that Collagen IV degradation in mutant tissues does not affect visibly niche signaling. Given the progressive loss of developing cysts in mutant germaria even in the presence of a normal pool of GSCs, we next performed a lineage experiment in which we compared the ability of control *versus* mutant germaria to produce new cysts. To this end, we generated flip-out clones to induce the expression of a reporter gene (*mCD8*::*GFP*) in random germline cysts from 1-week old ovaries. Females of this age were chosen because the vast majority of mutant ovaries this old do not present gross morphological alterations that might cause pleiotropic effects on cyst production (see above). We scored the number of germaria containing GFP-expressing cysts 3 days after clone induction to make sure they were generated from labeled GSCs or cystoblasts. We found marked germline cysts in 18.5% of control ovarioles (n = 216) and in 10.4% of mutant ones (n = 202). Normalized by the total number of GSCs in each of the groups, we found a frequency of 6.6 marked ovarioles per 100 GSCs in control ovaries, while mutant ovaries contained on average 4 marked ovarioles per 100 GSCs (S4 Table). Altogether, the above results demonstrate that 1-week old *timp* mutant germaria are only 60.7% efficient in germline cyst production compared to controls of the same age and they strongly support the idea of a direct correlation between *timp* activity and tissue homeostasis.

### *timp* regulates Mmp1 and Mmp2 distribution

**Fig 7:**
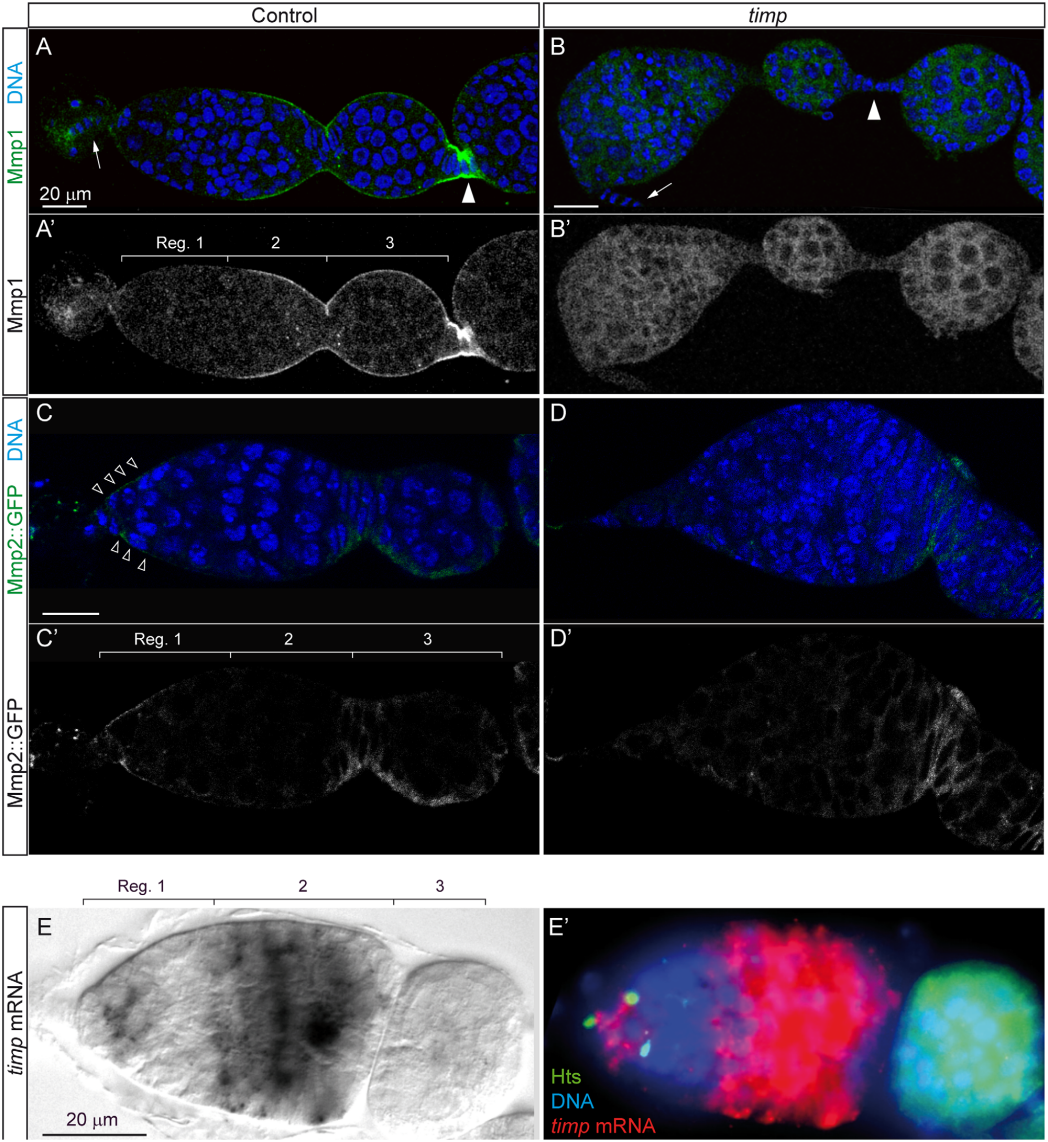
Mmp1 and Mmp2 are abnormally distributed in *timp* mutant ovaries. (A, A’) Pattern of Mmp1 accumulation in control ovaries. (B, B’) *timp* null mutant ovaries (*timp*^*28*^/Df ED5472) displayed a diffused Mmp1 staining, particularly in the stalks. (C, C’) Mmp2::EGFP is localized to the anterior half of the germarium in control ovaries. (D, D’) Mmp2::EGFP localization at the anterior tip of *timp* mutant germaria was not detected. Arrows: terminal filament. Arrowheads: interfollicular stalk. (E, E’) Double in situ hybridization and antibody staining to visualize the pattern of expression of *timp* mRNA in a wild-type germarium (E and red signal in E’) and the spectrosome marker Hts (green in E’). DNA was counterstained in blue. *timp* was strongly expressed at the tip of region 1, were GSCs and cystoblasts reside, and in region 2. Images can be composites of several focal planes.

Because the canonical role for *Drosophila timp* is to regulate MMP activity in a variety of tissues, we sought to determine if MMP distribution was affected in mutant ovaries. Mmp1 localizes to the terminal filament and in the cap cells area in control ovaries. From region 2 onwards it accumulates evenly along the basal side of the follicle cells. Stronger signal can be detected in the interfollicular stalks of stage 2 and older egg chambers. In striking contrast, null *timp* mutant germaria and interfollicular stalk cells show a diffused distribution of Mmp1 protein (Fig 7A and 7B). An Mmp2::GFP fusion protein driven by its endogenous promoter has been reported to accumulate most strongly in the anterior tip of the germarium, where it mediates the distribution of the niche signal Wingless [10]. Upon examination of the localization of Mmp2::GFP in mutant germaria, we found that *timp* mutants do not show a conspicuous signal in the niche region, indicating that, like in the case of Mmp1, *timp* activity affects Mmp2 localization (Fig 7C and 7D). This view is supported by the fact that *timp* mRNA is expressed strongly in the area were GSCs reside and in region 2 (Fig 7E and 7F). Interestingly, the expression domains of Mmp1 and Mmp2 in the germarium and of Mmp1 in the interfollicular stalks correlate with the *timp* mutant phenotypes described above and suggest a possible role for *timp* in regulating MMP activity in the germarium. Because the germarium and interfollicular stalk phenotypes are rescued by the ectopic expression of *timp* (see Materials and Methods and S2 Table), our observations suggest that endogenous *timp* might antagonize Mmp activity that to ensure germarium organization and proper interfollicular stalk formation.

## Discussion

In this work, we have shown that *Drosophila timp* is required for normal ECM metabolism in the ovary and that the loss of *timp* affects the physical properties, organization and homeostasis of the germarium. Understanding the link between ECM integrity and niche function is of vital importance considering that the ECM is an essential component of vertebrate stem cell niches [2, 21, 31]. Our findings provide a novel link between ECM integrity and niche organization and activity of potential importance for more complex niches.

### *timp* regulates Collagen IV turnover in the *Drosophila* ovary

Most evidence from *Drosophila* has reinforced the canonical view of MMPs and Timp as opposing regulators of ECM turnover, especially in tissue remodeling [28, 32]. For example, in a number of different *Drosophila* tissues ectopic *timp* expression phenocopies the loss of MMPs [33–35] and the phenotypes of the sub-viable *timp* mutant adults used in this work, such as wing blisters [12], are strongly suggestive of perturbed ECM regulation. However, up to now little or no direct evidence has been presented for the roles of endogenous *timp* in *Drosophila* or how its activity impinges on the regulation of ECM turnover. In this work we present several lines of evidence that support a canonical role for Timp in regulating ECM turnover in the *Drosophila* ovary. The reduced levels of the two Collagen IV subunits in *timp* mutant ovaries are consistent with an alteration in the turnover of this conserved basement membrane component [36]. Collagen IV is composed of long helical trimers that form a network via end domain and lateral interactions [37]. Significantly, because the increased proteolytic cleavage of human Collagen IV-FITC by cultured *timp* mutant ovaries could be blocked using a general MMP inhibitor, our observations strongly suggest that the loss of *timp* alters Collagen IV turnover via changes to MMP proteolytic activity. *Drosophila* embryos with reduced MMP activity showed lower levels of Collagen IV accumulation at the leading edge of epidermis wound sites [38]. Because Mmp1 and 2 act redundantly to promote wound healing, which could be blocked by overexpressing *timp*, MMP activity is required for Collagen IV deposition at least in the embryo. Our results indicate that during oogenesis endogenous *timp* activity is required for normal ECM organization, most likely by maintaining an appropriate balance of extracellular protease activity, a suggestion in agreement with the fact that *timp* overexpression blocks MMP2-dependent posterior follicle cell trimming during ovulation [35]. Further investigation will be required to confirm if one or both of the *Drosophila* MMPs are required for Collagen IV remodeling in oogenesis. However, it is also possible that *timp* controls ECM composition in other ways, as MMP-independent functions have been reported for *timp* in other organisms (reviewed in [39]).

### *timp* determines the mechanical properties of the ovarian niche

Given the altered Collagen IV levels in *timp* mutants and the morphological changes to ovariole organization, we expected to see alterations to ECM composition and gross basement membrane lesions. Surprisingly, we found that the distribution of conserved basement membrane components was not consistently affected when examined by confocal microscopy. Nor did we observe significant alterations to the strikingly electron dense extracellular matrix accumulations seen in the apical stem cell niche regions of the germarium. Atomic Force Microscopy has proved useful for studying the regulation of ECM molecules by extracellular proteases in other models (reviewed in [40]). Our examination of the mechanical properties of control and *timp* mutant ovaries using this method demonstrated that germaria and egg chambers were significantly softer than control tissues. While ovarioles are composite materials with different structures contributing to the measured apparent elastic modulus (*K)*, structures closer to the surface (such as the basement membrane) will contribute more than internal components. In fact, structures further away from the surface than ~1/10 of the indentation depth will not add to *K* [41, 42]. Given the ~100nm thickness of most ovarian basement membranes, the dramatic reduction in tissue stiffness of *timp* mutants in the 200nm indentation measurements is almost certainly a consequence of changes to the basement membrane itself. Under normal circumstances and considering the role of Collagen in determining a tissue’s elastic stiffness [21], the consistently Collagen IV-rich ovarian basement membranes are likely to form a stiff layer. This conserved ECM component has been shown to promote the basement membrane-mediated constriction of *Drosophila* tissues and organs [43]. Overall our data strongly suggest that in the absence of *timp* the ECM surrounding ovarian tissues, and particularly the germarium, has altered mechanical properties conferring lower stiffness despite a largely normal distribution of major ECM components.

The nature of ECM changes in *timp* mutants remains to be determined. It could reflect alterations to the molecular organization of ECM components such as Collagen IV and Laminin that are not visible to confocal or electron microscopy. Although we have tested the distribution of many different ECM components in *Drosophila*, it was not possible to examine the distribution of all the 20+ known or putative ECM components encoded by the *Drosophila* genome [44]. Developing suitable tools to determine the distribution of these additional ECM components will be needed to fully evaluate the role of *timp* in determining ECM composition. Of particular interest will be the remaining uncharacterized putative ECM components, such as Glutactin [45], that were detected by the iTRAQ screen as being expressed in the ovary.

### Is MMP activity in the ovary controlled by redundant mechanisms?

The relatively normal distribution of ECM components in *timp* mutant ovaries is related to another key question: how do adult flies develop and survive for weeks in the absence of *timp*? What stops secreted and membrane-bound MMP proteins from wreaking havoc in the absence of Timp? Mouse knockouts of individual *timp* genes are viable [46–49] and often fertile, exhibiting relatively minor defects in specific tissues frequently alleviated by decreasing MMP function in the affected tissues (reviewed by [50]. There are some interesting parallels between the effects of removing *timp* from mice and *Drosophila*. For example, mice null for *timp-3*, the closest orthologue to *Drosophila timp* [11], are viable but show decreasing lung function (and increasing collagen proteolysis) with age that eventually results in death after around 13 months [48].

MMP activity is believed to be tightly regulated at multiple levels including transcription, secretion and zymogenic-activation [51]. Our observation that MMP accumulation in the basement membrane is reduced in *timp* mutants suggests that the expression of *Mmp1* and *Mmp2* might be regulated to compensate for the absence of *timp*. In other *Drosophila* tissues, it has been shown that the JNK pathway controls *Mmp1* expression [34, 52]. We hypothesize the existence of feedback mechanisms in the ovary that detect the level of extracellular proteolytic activity and adjust *MMP* expression accordingly, a mechanism that may alleviate the absence of *timp* function in younger mutant females but that is insufficient to compensate the prolonged loss of *timp* in aged ovaries. The eventual defects seen in *timp* mutants as they age might result from less effective tissue homeostasis caused by the long-term absence of Timp protein or by reduced *MMP* expression.

### The role of *timp* in the ovarian stem cell niche: a close link with MMP expression

The germarium houses germline (GSC) and follicle stem cell (FSC) populations that give rise to the germline and somatic components, respectively, of developing egg chambers. Cap cells, escort cells and terminal filament cells are responsible for producing niche signals that permit the maintenance of GSCs in their undifferentiated state [19]. Here, we have shown that the loss of *timp* can severely alter the shape and organization of the different domains of the germarium as mutant flies age and enlarge interfollicular stalks between egg chambers. Over-expression of *timp* in the germarium produced the opposite effects with elongated compound germaria and egg chamber fusions. In spite of the often-dramatic changes to germaria morphology seen in *timp* mutants, the basic association of GSC, cap cells and the terminal filament was always intact. This is consistent with our observation that GSC number is unaffected in *timp* mutants and suggests that E-cadherin-mediated adhesion between GSCs and cap cells [53] maybe sufficient to maintain local GSC niche integrity even if ECM properties are compromised. Thus *timp* appears to be important for the long-term maintenance of germarial structure and its stereotyped arrangement of different subdomains.

FSC maintenance and proliferation in the niche depends on binding Laminin that they themselves secrete [54]. In addition to providing structural support within stem cell niches, the ECM is also believed to play a key role in stem cell niche signaling [2, 21]. Recently, it has been shown that the secreted glypican Division abnormally delayed (Dally)-like (Dlp) promotes the long-range action of the Wingless (Wg) ligand in the germarium. Mmp2, which can cleave Dlp in cell culture, opposes Dlp activity in the ovary, thus limiting the range of Wg signaling. Interestingly, the phenotypes associated with loss- and gain-of-function conditions for Mmp2 are similar to those we identified for *timp*, with long stalks seen in conditional *Mmp2* mutants and egg chamber fusions when overexpressed [10]. These results are in accordance with our finding that loss of *timp* results in decreased Mmp2 accumulation in the anterior tip of the germarium and suggest that *timp* activity may regulate FSC proliferation via Mmp2. In support of this idea, ectopic *timp* overexpression suppressed both the long stalks of *Mmp2* mutants and egg chamber fusions of *Mmp2* overexpression [10].

We envisage the basement membrane as a physical corset responsible for the maintenance of the proper shape of the ovarian niche, in spite of the tensions generated by the cell divisions and cellular movements that occur during new egg chamber assembly. This corset could be analogous to the Collagen IV—dependent structure that controls follicle shape during of the later stages of oogenesis [14]. The circumferential migration of FSC daughter cells [55] may play a role in secreting and/or organizing such a circular structure. Considering the striking defects observed in the organization of the mutant ovarioles, in which germaria displayed abnormal shapes and an aberrant arrangement of cell types, we propose that the loss of Timp regulation of the ECM causes a softening of the basement membrane, which is now unable to act as the corset upon which the tissue is modeled. As a consequence and as indicated by the progressive loss of developing cysts in aging mutant germaria, the new arrangement of the GSC and FSC niches provokes a significant impairment of ovarian homeostasis. Because a large number of stem cell niches of both vertebrates and invertebrates have been found to be contained within, or to interact with, specialized ECMs, the findings reported here provide a link between ECM metabolism, niche organization and tissue homeostasis that may be of general importance for the biology of stem cells.

## Materials and Methods

(See the Supporting S1 Text for detailed procedures)

### Fly stocks

We generated a *timp* null condition by combining a ∼15 kb deletion of that removes both *timp* and *synapsin* (*timp*^*28*^, a gift from A. Page-McCaw) with the larger Df(3R)ED5472 (Bloomington *Drosophila* Stock Centre). Females bearing a deletion only affecting *synapsin* (*syn*^*27*^) [12] over Df(3R)ED5472 display none of the phenotypes associated with the removal of *timp*. The presence of a *UASt-timp* and either *Heat-Shock Gal4* or the *c587-Gal4* transgenes in *timp*^*28*^/Df(3R)ED5472 mutant females grown at 25°C was sufficient to significantly restore normal ovary morphology. Viking: GFP is a protein trap in the endogenous Collagen IV α2-chain (line G00205; FlyTrap; http://flytrap.med.yale.edu/). *UASt-timp*, a gift from A. Page-McCaw, is a pUASt insertion carrying the complete *timp* coding sequence [28]. *c587-Gal4* is a germarium-specific GAL4 line [29]. Mmp2-EGFP is an engineered BAC construct (*P[acman]-Mmp2-EGFP-GPI*; [10] that expresses EGFP-tagged Mmp2 under endogenous regulatory sequences.

To perform the flip-out experiments, we obtained *w*, *hsFLP12/w; 10xUASt-mCD8*::*GFP/+; timp*^*28*^ *Act>y*^+^*>Gal4*/TM6B and *w*, *hsFLP12/w; 10xUASt-mCD8*::*GFP/+; timp*^*28*^ *Act>y*^+^*>Gal4*/*Df(3R)ED5472* genotypes. Control and experimental flies were heat-shocked at 37°C for 30 minutes and the ovaries dissected and processed for immunostaining 3 days later.

### Preparation of fixed ovaries for observation. Antibodies used

Antibody, DNA and rhodamine-phalloidin stainings were performed according to standard procedures. A list of the antibodies used is available in the Supporting S1 Text.

### Preparation of live ovaries for observation

Individual ovarioles were dissected in Schneider medium supplemented with streptomycin and insulin as described previously [56].

Fluorescence Recovery After Photobleaching (FRAP)

Regions of interest were bleached at 100% 488 nm laser power for three 2-second scans (400 Hz, one line/frame average). Images were then collected at 20-minute intervals for 2 hours.

### Quantification of fluorescence

Image series were captured using identical confocal settings for control and experimental ovarioles. Color depth was set to 12-bit and configured to minimize saturated pixels. Depth (z) thresholds were set well above and below each ovariole to guarantee the complete tissue was captured. Sections were taken at 630 nm intervals (optimal). FITC was captured using the default Leica FITC configuration and low laser intensity. After recording position and z-depth ranges, image series were captured automatically. All image stacks were pre-processed using the standard background subtraction function of ImageJ (default settings; 50 pixel radius). Measurements were taken using the Imaris *Measurement Points Tool* along the length of each ovariole (4 in the terminal filament, 5 in regions 1-2a of the germarium, 3 in regions 2b-3, 4 in each of the stalks and 4 measurements in each of the egg chambers). Each ovariole contained at least 3 stalks and 4 egg chambers. Ovarioles were measured along three different lines running from the germarium to posterior follicles. Thus, TFs were quantified 12 times/ovariole, the niche region 6 times/ovariole, etc.

### Transmission Electron Microscopy (TEM)

TEM samples were prepared following standard procedures. Sections were examined with a Zeiss EM902 electron microscope at 80Kv, and photographed at 50.000x magnification.

### Collection of *Drosophila* ovaries for proteome analysis

0–3 day-old females were yeasted for 3 days prior to dissection. Ovaries were dissected in PBS and immediately frozen in liquid nitrogen. For the iTRAQ experiment, we collected four biological replicas. For the LTQ-Orbitrap, we analyzed two biological replicas. For the 2D DIGE analysis, we collected four biological replicas.

### iTRAQ quantitation

Protein quantitation was carried using four independently-collected samples from control and experimental ovaries. A MALDI TOF/TOF 4800 (AB SCIEX, Foster City, CA, USA) mass spectrometer was used for acquisition and data processing. An overview of the experimental design is shown in Fig 1B. The final quantitation was performed on identified proteins associated at least to three quantitated peptides.

### LC-MS/MS analysis of wild-type ovaries on a LTQ-Orbitrap

Prepared protein samples were digested with 1:20 sequencing grade trypsin (Roche Molecular Biochemicals). Peptides were analyzed using a linear trap Orbitrap Velos (LTQ Orbitrap Velos) hybrid mass spectrometer. The identified fragments were searched against the *Drosophila melanogaster* database of UniProtKB/Swiss-Prot using Mascot (version 2.3.0).

### DIGE analysis

A total of 50 μg of proteins from each condition labeled with 400 pmol of Cy3 or Cy5 dyes were used to perform Isoelectro focusing. The second dimension was performed on 12% SDS-PAGE gels. Fluorescent gel images were scanned and changed protein spots with p-values ≤0.05 were picked for finger-printing identification. MALDI samples were automatically analyzed in an Ultraflex MALDI-TOF/TOF mass spectrometer.

### GO analysis of the differentially expressed proteins

To perform ontological and functional studies, a list of candidate genes coding for differentially-expressed proteins was evaluated using PANTHER (http://www.pantherdb.org/) and GeneCodis (version 3; http://genecodis.cnb.csic.es/) tools.

### Zymography assays

0–3 day-old females were yeasted for 3 days prior to manipulation. To assay for the collagen IV degradation in live tissue, we dissected control and experimental ovaries in Schneider’s medium supplemented with Fetal Bovine Serum (15% vol/vol) containing streptomycin/penicillin [56] and incubated them in a culturing cocktail containing Collagen IV-FITC (Collagen, type IV from human placenta, fluorescein conjugate; Invitrogen). Upon fixation in 4% paraformaldehyde, the fluorescence of control and experimental samples were captured using identical confocal settings and measured using IMARIS software.

### Atomic Force Microscopy analysis

137 measurements from 7 *ex-vivo* wild-type ovarioles and 179 from 12 *timp* nulls were collected. Monodisperse polystyrene beads were glued to silicon cantilevers with a nominal spring constant of 0.1 N/m. Force-distance-curves were taken at an approach speed of 10μm/s and a maximum force *F* = 6nN. Force—distance curves were analyzed for different indentation depths δ (0.2μm, 0.5μm and 1μm) using a Matlab-based custom algorithm [57].

## Supporting Information

S1 Fig LTQ-Orbitrap analysis of wild-type ovaries confirms a shared proteome with *ovo*^*D1*^ ovaries. (A) Relative sizes of ovaries from 5-day old females of the following genotypes: *timp*^*28*^/TM3, controls. *timp*^*28*^/Df ED5472, *timp* mutants. *ovo*^*D1*^/+;; *timp*^*28*^/TM3 controls (iTRAQ). *ovo*^*D1*^/+;; *timp*^*28*^/Df ED5472, mutant ovaries (iTRAQ). While the *ovo*^*D1*^mutation largely blocks oogenesis at stage 4 approximately, we could observe—more often in the *ovo*^*D1*^/+;; *timp*^*28*^/TM3 genetic background than in the *ovo*^*D1*^/+;; *timp*^*28*^/Df combination—a few escaper mature follicles within the dissected ovaries, which may account for the vitellogenic proteins found in the iTRAQ experiment. (B-D) In order to test whether the ovaries used in the iTRAQ experiment contained a representative proteome of the normal tissue, we used an LTQ-Orbitrap ion trap mass spectrometer to compare a fraction of the proteome of *w*^*1118*^ (wild-type) ovaries with that of *ovo*^*D1*^/+;; *timp*^*28*^/TM3, as determined by the iTRAQ study. The LTQ-Orbitrap analysis identified 610 proteins with a MASCOT score above 50 and a peptide hit ≥ 2.380 proteins (62.3%) were also found in the *ovo*^*D1*^/+;; *timp*^*28*^/TM3 iTRAQ analysis (S1 Table). A Gene Ontology analysis utilizing the GeneCodis tool to search for biological annotations significantly associated to both sets of identified genes rendered similar results, with over 90% of the clustered protein hits in the iTRAQ and LTQ approaches falling in the same Biological Process categories. Boxes outlined in grey denote categories not common to both data sets. Scale bar: 500 μm.
(TIF)

S2 Fig 2D-DIGE validation of identified candidates. (A-C) With the aim of validating some of the candidates over-represented either in control (*w*^*1118*^;; *timp*^*28*^/TM3) or in experimental ovaries (*w*^*1118*^;; *timp*^*28*^/D(3R) ED5472), we performed a two-dimensional DIfference Gel Electrophoresis (2D-DIGE). Protein samples isolated from both types of ovaries were labeled with different fluorescent dyes and separated in two-dimensional electrophoresis. This global approach allowed the isolation and identification by mass-spectrometry of three proteins. Two of these proteins were up-regulated in control samples (Chorion protein S16 and Vitelline membrane 26Aa, with standardized logarithm abundance ratios of 3.02 and 3.67, respectively) and the third one was expressed in *timp* mutant ovaries at higher levels (Regucalcin, average ratio of -1.56) (S1 Table). In agreement with these findings, the iTRAQ experiment identified another of the chorion proteins in the *Drosophila* genome (S15) as over-represented in control ovaries, while Regucalcin was found over-expressed in mutant ovaries (S2 Table).
(TIF)

S3 Fig Collagen IV-FITC degradation is Mmp-dependent. Confocal images of control egg chambers (*timp*^*28*^/TM3) after incubation in culture medium with Collagen IV-FITC for two hours. (A) Without inhibitor treatment. FITC signal. (B, B’) Pre-incubated for 30 minutes with 1mM 1, 10-Phenanthroline, a general inhibitor of MMP activity. FITC and transmission channels.
(TIF)

S4 Fig The distribution of core ECM constituents in the ovarian basement membrane is not visibly affected in *timp* mutant females. Confocal images from 2-week old control (*timp*^*28*^/TM3) and mutant (*timp*^*28*^/Df ED5472) ovarioles. (A, B, E-H, K-N) Confocal cross-sections of fixed tissue. (C, D) Collagen IV:GFP expression in living egg chambers. (J, K) Perlecan:GFP expression in living egg chambers. Images are z-projections of several confocal sections of the basement membrane. Note the orientation of Collagen IV and Perlecan fibrils perpendicular to the axis of rotation. Confocal images can be composites of several focal planes. In all panels anterior is to the left. Scale bars: 25 μm.
(TIF)

S5 Fig Quantification of core ECM constituents in the ovarian basement membrane. Graphical representation of the values for Laminin-γ, Laminin-β, Col IV α2 and Perlecan. Fluorescence intensities of confocal micrographs captured under the same conditions and treated in parallel were compared. Between 8 and 12 ovarioles were scored in 7 or 8 areas along their anterior-posterior axis (germarium tip or region 1, regions 2–3, egg chambers (EC) 1 to 4, and first interfollicular stalk (stalk 1)). Each measurement contained 8 pixels and the intensity of the signal was determined using the Maximum intensity value. Our results confirm that there are no consistent changes between Perlecan, Col IV, Laminin-gamma and Laminin-beta distribution in controls and *timp* mutants. Genotypes: control (*timp*^*28*^/TM3) and mutant (*timp*^*28*^/Df ED5472). *p* values of two-tailed t-tests *<0.05, **<0.005, ***<0.001.
(TIF)

S6 Fig Transmission electron microscopy analysis of *timp* ovaries. TEM images of germaria from control and mutant ovaries. (A, B) GSC niche region of control and mutant germaria, respectively. For reference, cap cells are pseudo-colored in red and GSCs in yellow. (C, D) Images of lateral sides (regions 2–3) of control and mutant gemaria. (E, F) Micrographs from 2-week old *timp* germaria showing the cellular degeneration characteristic of mutant ovaries. In addition to the clear cytoplasms present in escort cells in (E, magnified in E’), mutant cells display multi-vesicular vacuoles containing cell debris (open arrowheads) and multi-lamellar bodies (asterisks). These are hardly seen in control tissues. Black arrowheads point at electron dense ECM material.
(TIF)

S7 Fig FRAP analysis of Collagen IV:GFP. Regions of interest in the basement membrane (BM) of control and experimental egg chambers were photobleached at three different time points in oogenesis, stages 1–3, 5–6 and 7–8. S4 follicles could not be staged unambiguously and were not included in the quantifications. Fluorescence recovery was quantified 0 and 100 min. after bleaching. (A) Collagen IV:GFP expression in a control egg chamber before and after bleaching. (B) Time-lapse images of control and experimental BMs showing Collagen IV:GFP fluorescence 0 and 100 minutes after photobleaching. S1-3 egg chambers from control or experimental ovaries did not recover Collagen IV:GFP fluorescence at significant levels. In contrast, both control and *timp* mutant S5-6 and S7-8 follicles showed an increase in fluorescence after 100 minutes. (C) Graph showing quantification of fluorescence recovery 100 min. after photobleaching. The average ± standard deviation values of fluorescent increments (in arbitrary units) are the following: S1-S3 controls, 0.21±0.36 (n = 5); S1-S3 mutants, 0.43±0.39 (n = 4); S5-S6 controls, 1.96±0.67 (n = 5); S5-S6 mutants, 1.22±1.05 (n = 5); S7-S8 controls, 8.95±5.64 (n = 5); S7-S8 mutants, 9.85±3.60 (n = 4). The average ± standard deviation values of rotation speeds (in microns/minute) are the following: S1-S3 controls, 0.15±0.01 (n = 2); S1-S3 mutants, 0.16±0.06 (n = 3); S5-S8 controls, 0.31±0.08 (n = 7); S5-S8 mutants, 0.30±0.09 (n = 5). The genotype of control and experimental flies is the following: *w*; *viking*:GFP/+; *timp*^*28*^/TM3 (control) and *w*; *viking*:GFP/+; *timp*^*28*^/Df(3R) ED5472 (*timp*). Images can be projections of several focal planes. *p* values of two-tailed t-tests *<0.05, **<0.005, ***<0.001.
(TIF)

S8 Fig Determination of ovariole tissue stiffness at different indentation depths. (A) Graph showing the stiffness of control ovarioles (*timp*^*28*^/TM3) at indentation depths of 0.2 μm, 0.5 μm and 1.0 μm. (B) Graph showing the stiffness of *timp* mutant ovarioles (*timp*^*28*^/Df ED5472) at the same indentation depths as above. (C) Graph displaying the relative changes in stiffness of control *versus* mutant ovarioles. In both wild-type and mutant ovarioles, we found highest stiffness in the first 0.2 μm to contact the probe and that greater indentation depths resulted in decreased overall stiffness. Note that mutant tissues are consistently softer that controls. *p* values of two-tailed t-tests comparing control and experimental measurements at 0.5 μm indentation depth were <0.01 in points 2, 3, 4, 5, 7 and 9, and <0.05 in points 6, 8 and 11. *p* values at 1 μm indentation depth were <0.01 in points 2, 3, 4, 5, 7, 9 and 11, and <0.05 in points 6 and 8. Image is a composite of several focal planes.
(TIF)

S1 Table Data sets of proteins and genes identified in the iTRAQ, LTQ and 2D-DIGE analyses. iTRAQ experiment: the Table contains the lists of identified and quantitated proteins and the ratios of relative quantities control/experimental for the Mascot search engine. The total average ratio was calculated as the geometric mean of at least two values from the search engine. The list of the 48 proteins found to be differentially expressed (i. e., with control/experimental average expression ratios ≤0.50 and ≥1.50) is shown, as well as the data for the peptides used in the quantitation. LTQ experiment: the Table includes the list of identified proteins with a peptide hit ≥ 2, High peptide confidence (p < 0.05) and a False Discovery Rate < 0.1. The MASCOT score was set above 50. 2D-DIGE: the Table shows the peptide sequences used to identify the spotted proteins, the MASCOT scores and the control/experimental average ratios.
(XLS)

S2 Table Quantification of germarium shape, terminal filament position and stalk cell phenotypes in controls, *timp* mutants and *timp* mutants carrying a *UASt-timp* transgene. Differences in length-to-width ratios between control and mutants are statistically significant (*p* value of two-tailed t-tests <0.001 for 14-day old ovaries). Please refer to Fig 6 for examples of the different phenotypic classes.
(DOCX)

S3 Table Quantification of the number of GSCs and cyst per germarium in controls and *timp* mutants. Differences in the #cysts/germarium between control and mutants are statistically significant (*p* values of two-tailed t-tests <0.001 for 2-, 10- and 21-day old germaria).
(DOCX)

S4 Table Quantification of cyst production in 1-week old controls and *timp* mutants. 1-week old adult females yeasted for 2 days were transferred to 37°C for 30 minutes to induce clone production. Ovaries were fixed, stained and prepared for observation 3 days later.
(DOCX)

S1 Text Supporting information. List of supporting figures and tables with additional experimental procedures for Fly Stocks, Preparation of fixed ovaries for observation/antibodies used, Preparation of live ovaries for observation, Transmission Electron Microscopy (TEM), Collection of Drosophila ovaries for proteome analysis, iTRAQ quantitation, LC-MS/MS Analysis, DIGE analysis, GO analysis of the differentially expressed proteins, Zymography assay and Atomic Force Microscopy analysis.
(DOCX)
