# Supplementary figures and images for: ECM-Regulator timp Is Required for Stem Cell Niche Organization and Cyst Production in the Drosophila Ovary
Source: PLoS Genet. 2016 Jan 25;12(1):e1005763. doi: 10.1371/journal.pgen.1005763 (PMC4725958; doi:10.1371/journal.pgen.1005763)

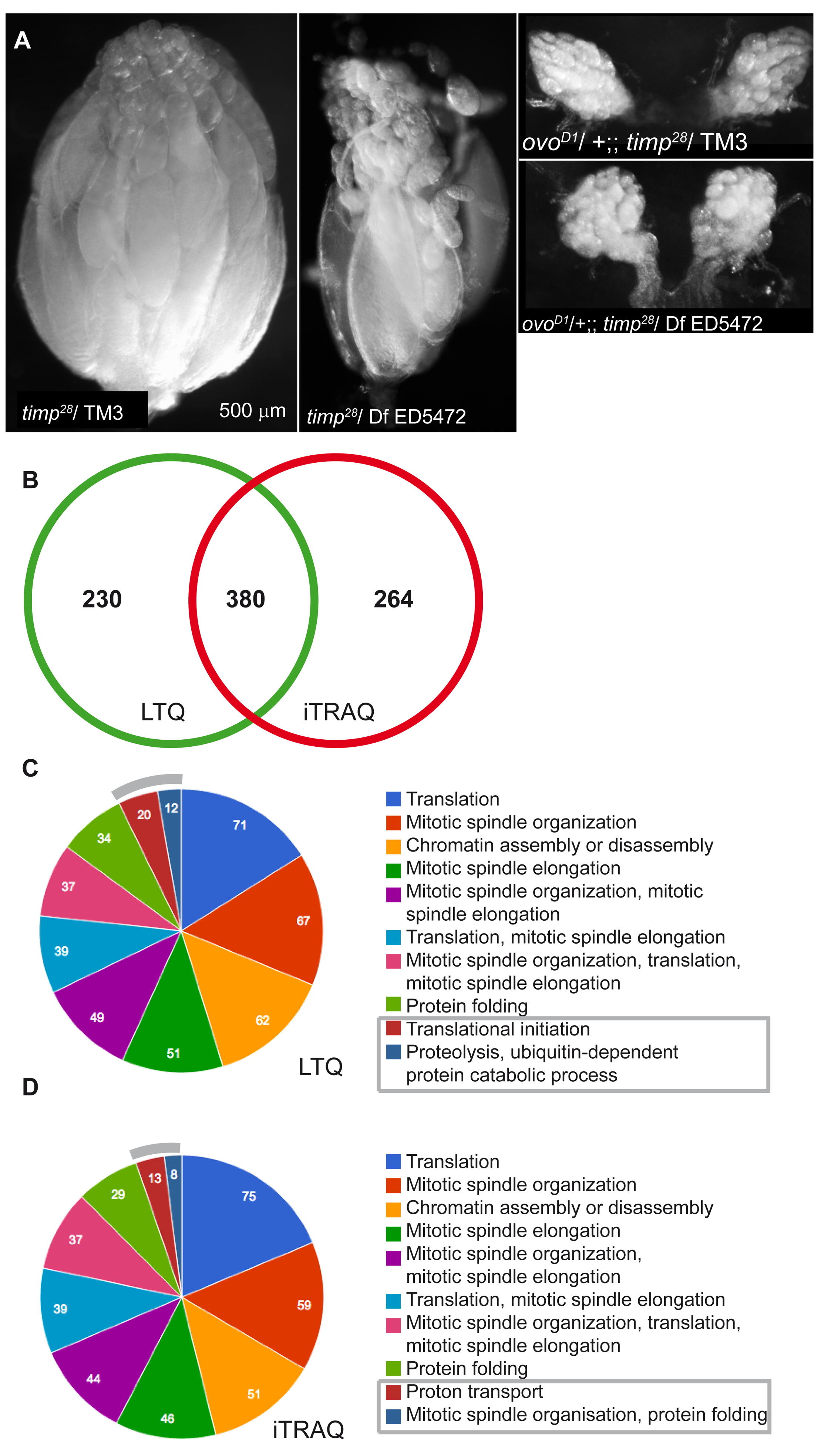

Supplement: S1 Fig — (A) Relative sizes of ovaries from 5-day old females of the following genotypes: timp28/TM3, controls. timp28/Df ED5472, timp mutants. ovoD1/+;; timp28/TM3 controls (iTRAQ). ovoD1/+;; timp28/Df ED5472, mutant ovaries (iTRAQ). While the ovoD1mutation largely blocks oogenesis at stage 4 approximately, we could observe—more often in the ovoD1/+;; timp28/TM3 genetic background than in the ovoD1/+;; timp28/Df combination—a few escaper mature follicles within the dissected ovaries, which may account for the vitellogenic proteins found in the iTRAQ experiment. (B-D) In order to test whether the ovaries used in the iTRAQ experiment contained a representative proteome of the normal tissue, we used an LTQ-Orbitrap ion trap mass spectrometer to compare a fraction of the proteome of w1118 (wild-type) ovaries with that of ovoD1/+;; timp28/TM3, as determined by the iTRAQ study. The LTQ-Orbitrap analysis identified 610 proteins with a MASCOT score above 50 and a peptide hit ≥ 2.380 proteins (62.3%) were also found in the ovoD1/+;; timp28/TM3 iTRAQ analysis (S1 Table). A Gene Ontology analysis utilizing the GeneCodis tool to search for biological annotations significantly associated to both sets of identified genes rendered similar results, with over 90% of the clustered protein hits in the iTRAQ and LTQ approaches falling in the same Biological Process categories. Boxes outlined in grey denote categories not common to both data sets. Scale bar: 500 μm. (TIF) [file pgen.1005763.s001.tif]

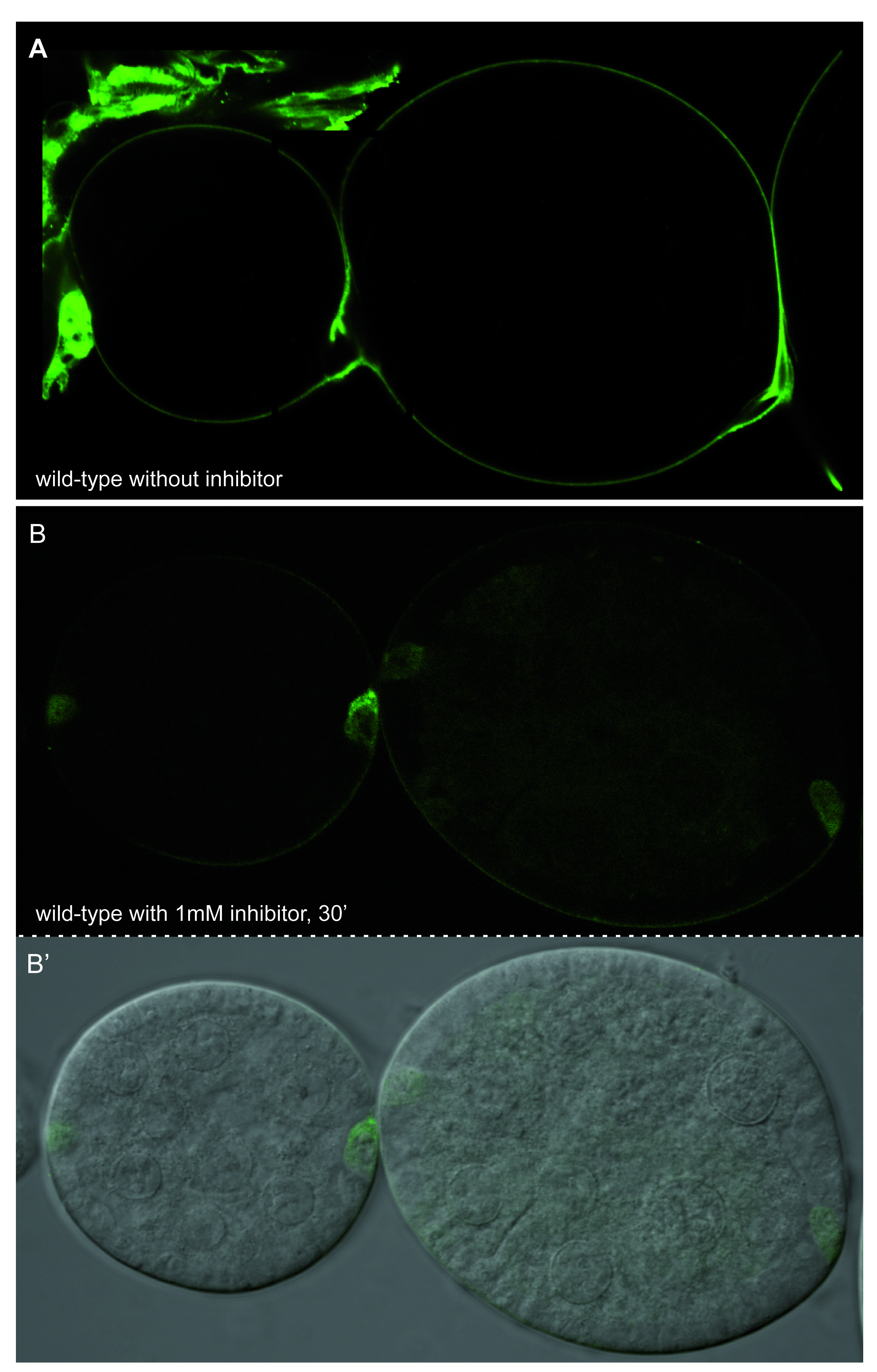

Supplement: S3 Fig — Confocal images of control egg chambers (timp28/TM3) after incubation in culture medium with Collagen IV-FITC for two hours. (A) Without inhibitor treatment. FITC signal. (B, B’) Pre-incubated for 30 minutes with 1mM 1, 10-Phenanthroline, a general inhibitor of MMP activity. FITC and transmission channels. (TIF) [file pgen.1005763.s003.tif]

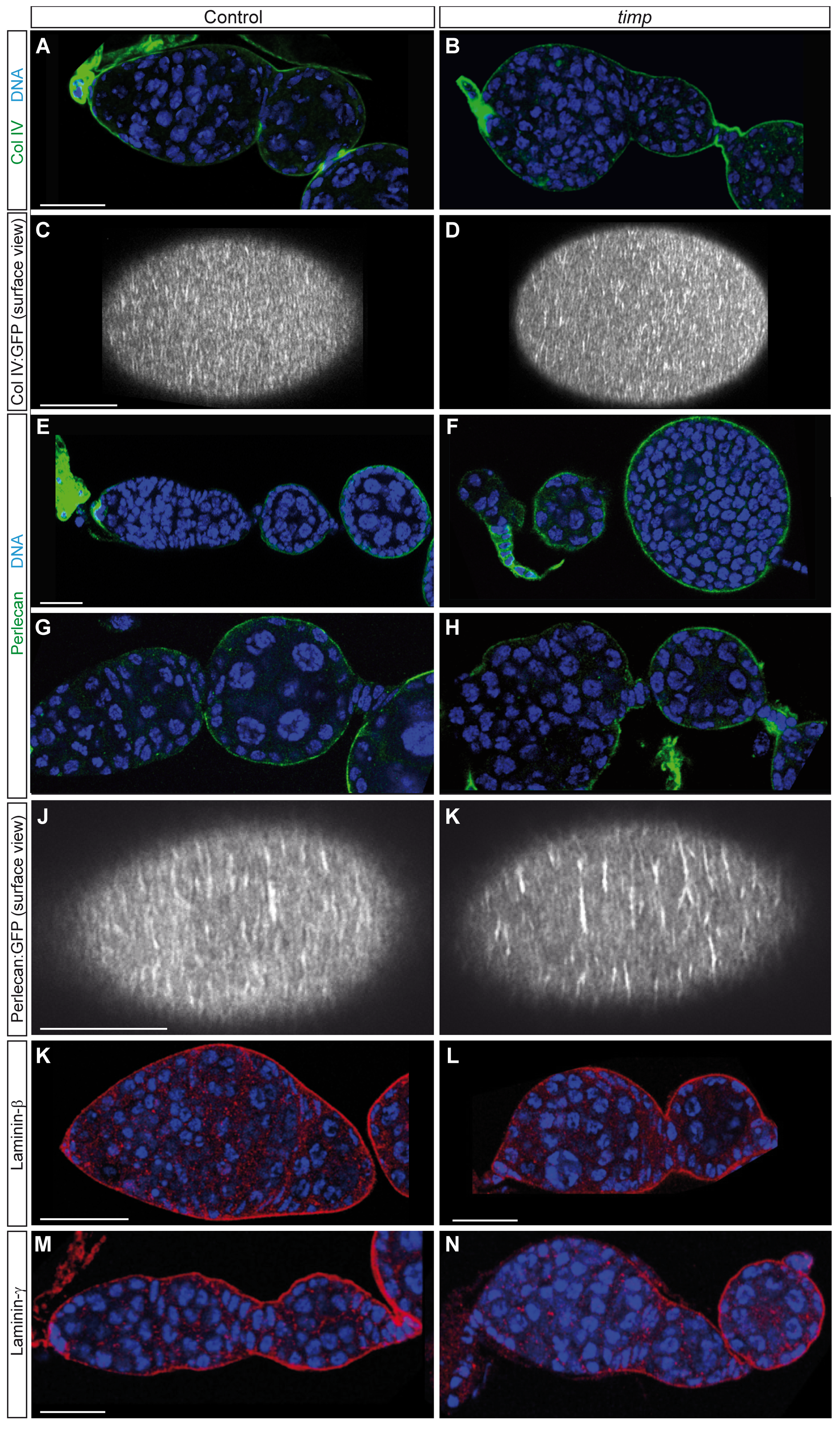

Supplement: S4 Fig — Confocal images from 2-week old control (timp28/TM3) and mutant (timp28/Df ED5472) ovarioles. (A, B, E-H, K-N) Confocal cross-sections of fixed tissue. (C, D) Collagen IV:GFP expression in living egg chambers. (J, K) Perlecan:GFP expression in living egg chambers. Images are z-projections of several confocal sections of the basement membrane. Note the orientation of Collagen IV and Perlecan fibrils perpendicular to the axis of rotation. Confocal images can be composites of several focal planes. In all panels anterior is to the left. Scale bars: 25 μm. (TIF) [file pgen.1005763.s004.tif]

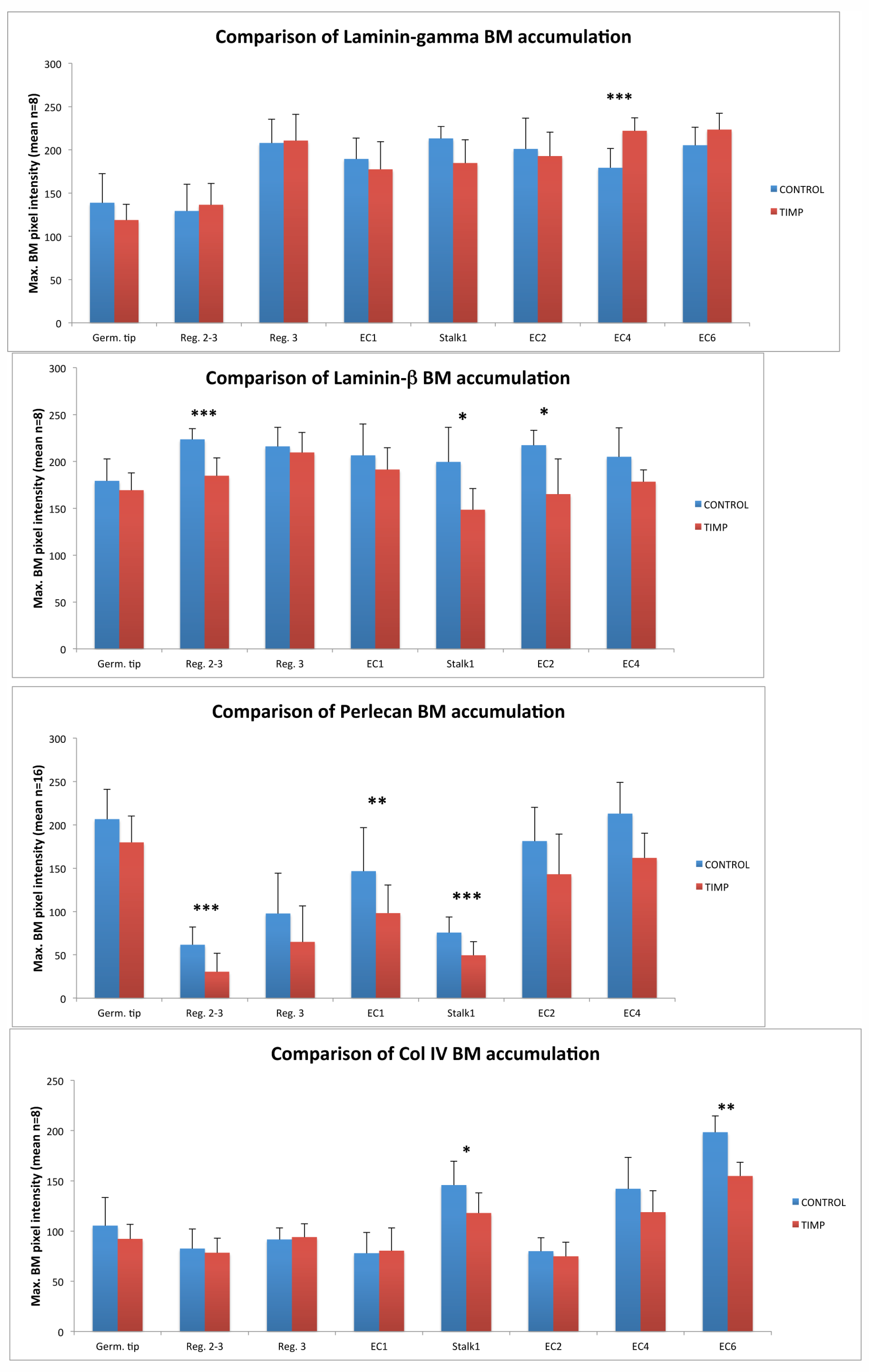

Supplement: S5 Fig — Graphical representation of the values for Laminin-γ, Laminin-β, Col IV α2 and Perlecan. Fluorescence intensities of confocal micrographs captured under the same conditions and treated in parallel were compared. Between 8 and 12 ovarioles were scored in 7 or 8 areas along their anterior-posterior axis (germarium tip or region 1, regions 2–3, egg chambers (EC) 1 to 4, and first interfollicular stalk (stalk 1)). Each measurement contained 8 pixels and the intensity of the signal was determined using the Maximum intensity value. Our results confirm that there are no consistent changes between Perlecan, Col IV, Laminin-gamma and Laminin-beta distribution in controls and timp mutants. Genotypes: control (timp28/TM3) and mutant (timp28/Df ED5472). p values of two-tailed t-tests *<0.05, **<0.005, ***<0.001. (TIF) [file pgen.1005763.s005.tif]

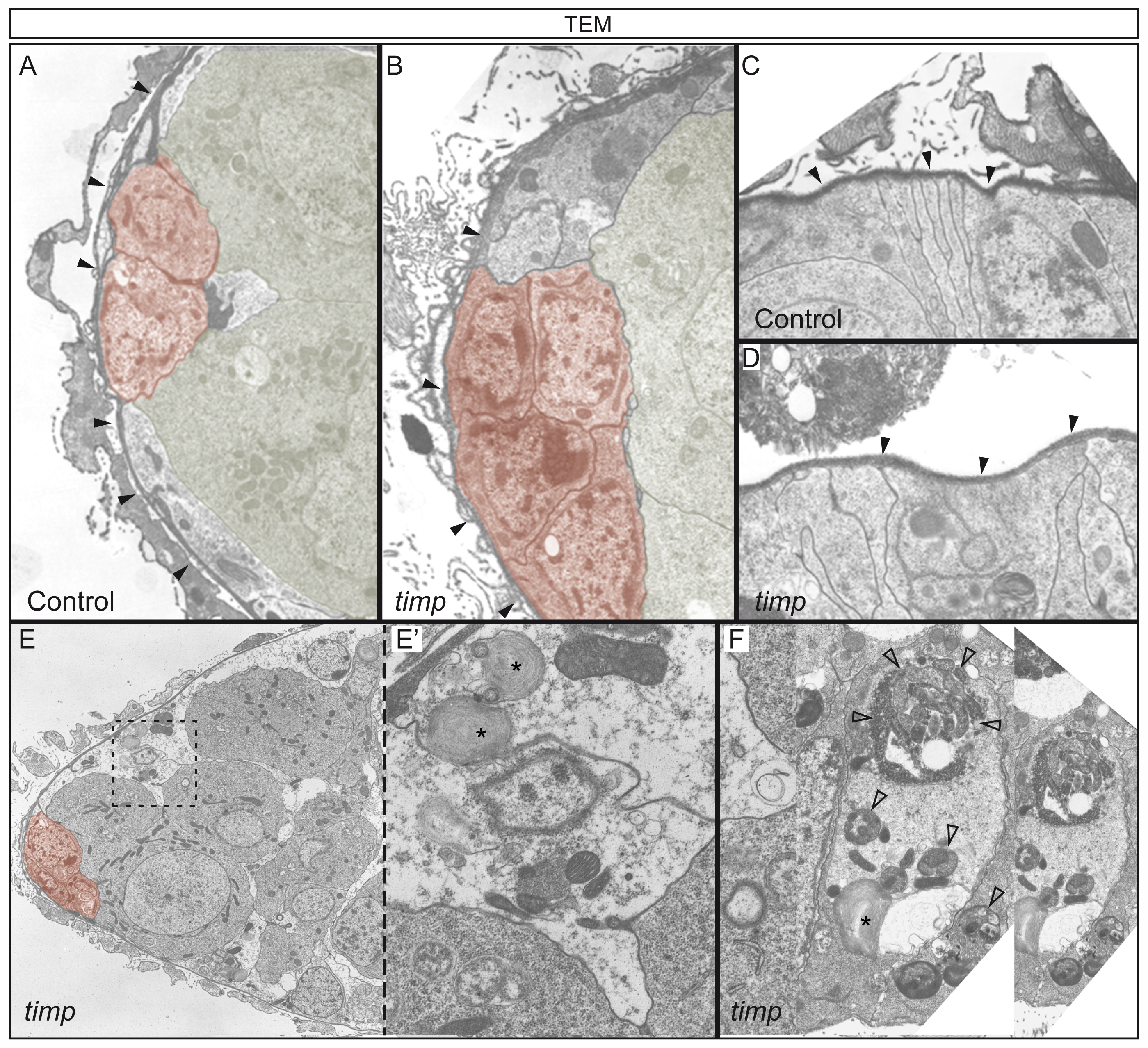

Supplement: S6 Fig — TEM images of germaria from control and mutant ovaries. (A, B) GSC niche region of control and mutant germaria, respectively. For reference, cap cells are pseudo-colored in red and GSCs in yellow. (C, D) Images of lateral sides (regions 2–3) of control and mutant gemaria. (E, F) Micrographs from 2-week old timp germaria showing the cellular degeneration characteristic of mutant ovaries. In addition to the clear cytoplasms present in escort cells in (E, magnified in E’), mutant cells display multi-vesicular vacuoles containing cell debris (open arrowheads) and multi-lamellar bodies (asterisks). These are hardly seen in control tissues. Black arrowheads point at electron dense ECM material. (TIF) [file pgen.1005763.s006.tif]

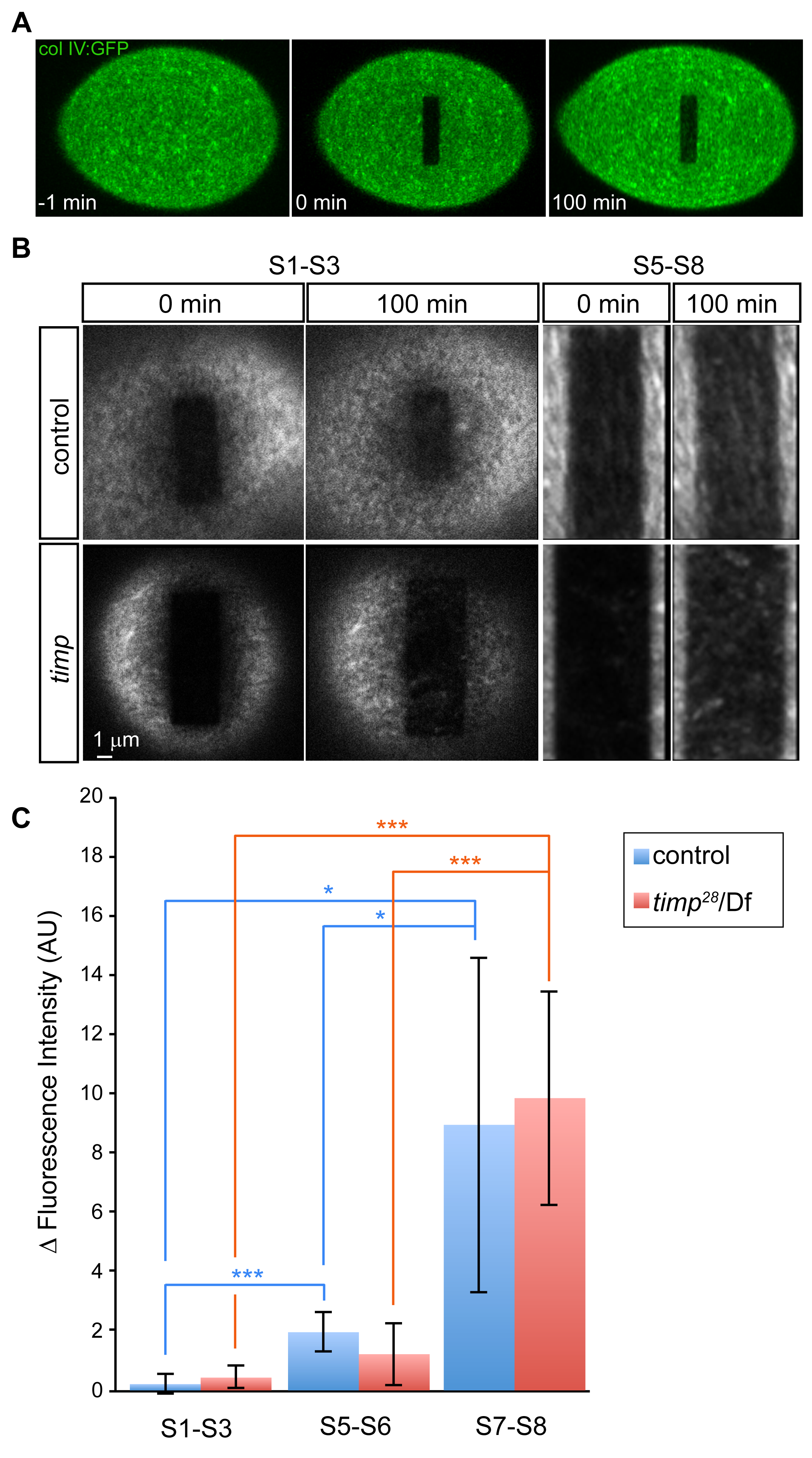

Supplement: S7 Fig — Regions of interest in the basement membrane (BM) of control and experimental egg chambers were photobleached at three different time points in oogenesis, stages 1–3, 5–6 and 7–8. S4 follicles could not be staged unambiguously and were not included in the quantifications. Fluorescence recovery was quantified 0 and 100 min. after bleaching. (A) Collagen IV:GFP expression in a control egg chamber before and after bleaching. (B) Time-lapse images of control and experimental BMs showing Collagen IV:GFP fluorescence 0 and 100 minutes after photobleaching. S1-3 egg chambers from control or experimental ovaries did not recover Collagen IV:GFP fluorescence at significant levels. In contrast, both control and timp mutant S5-6 and S7-8 follicles showed an increase in fluorescence after 100 minutes. (C) Graph showing quantification of fluorescence recovery 100 min. after photobleaching. The average ± standard deviation values of fluorescent increments (in arbitrary units) are the following: S1-S3 controls, 0.21±0.36 (n = 5); S1-S3 mutants, 0.43±0.39 (n = 4); S5-S6 controls, 1.96±0.67 (n = 5); S5-S6 mutants, 1.22±1.05 (n = 5); S7-S8 controls, 8.95±5.64 (n = 5); S7-S8 mutants, 9.85±3.60 (n = 4). The average ± standard deviation values of rotation speeds (in microns/minute) are the following: S1-S3 controls, 0.15±0.01 (n = 2); S1-S3 mutants, 0.16±0.06 (n = 3); S5-S8 controls, 0.31±0.08 (n = 7); S5-S8 mutants, 0.30±0.09 (n = 5). The genotype of control and experimental flies is the following: w; viking:GFP/+; timp28/TM3 (control) and w; viking:GFP/+; timp28/Df(3R) ED5472 (timp). Images can be projections of several focal planes. p values of two-tailed t-tests *<0.05, **<0.005, ***<0.001. (TIF) [file pgen.1005763.s007.tif]

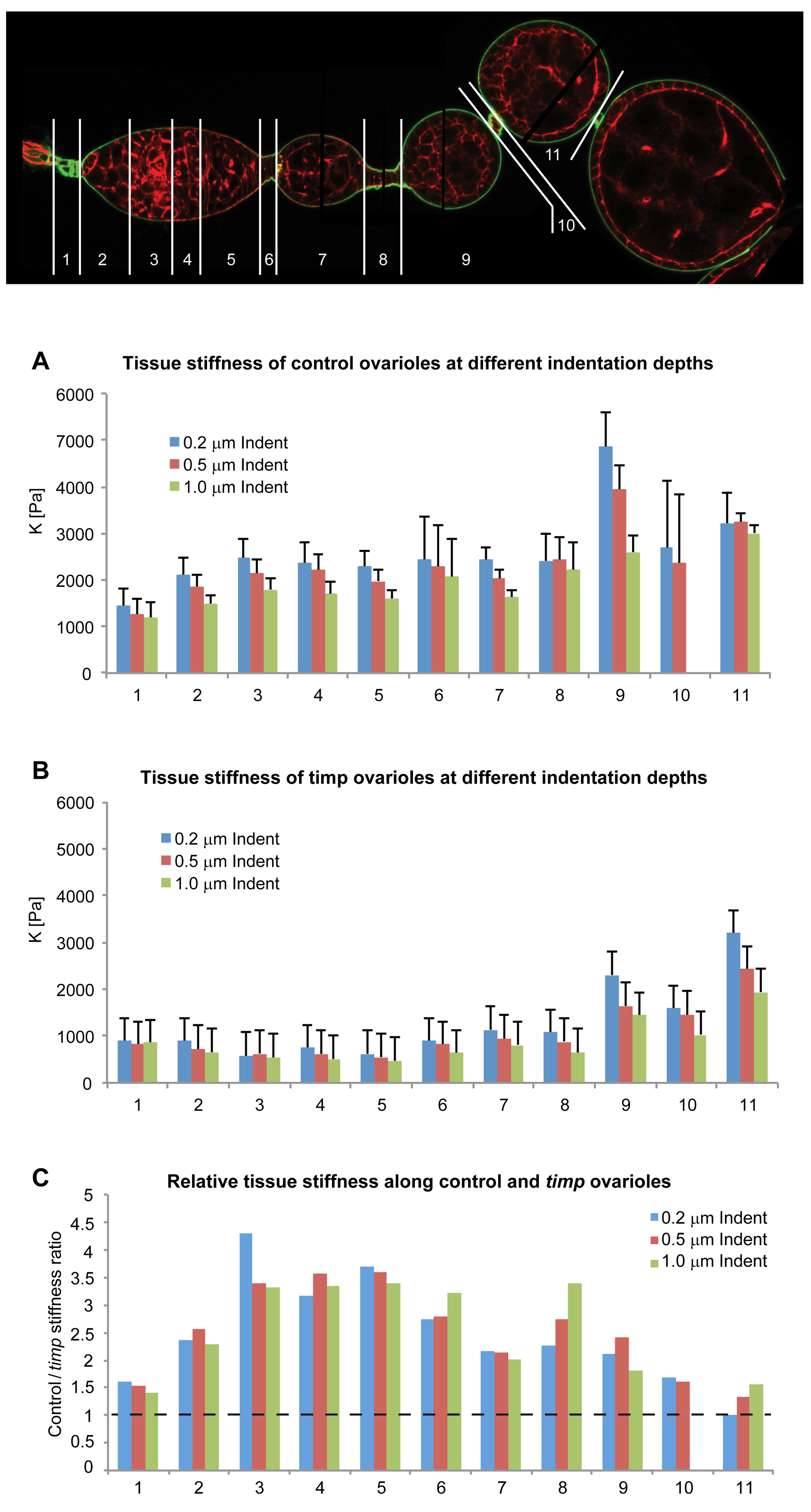

Supplement: S8 Fig — (A) Graph showing the stiffness of control ovarioles (timp28/TM3) at indentation depths of 0.2 μm, 0.5 μm and 1.0 μm. (B) Graph showing the stiffness of timp mutant ovarioles (timp28/Df ED5472) at the same indentation depths as above. (C) Graph displaying the relative changes in stiffness of control versus mutant ovarioles. In both wild-type and mutant ovarioles, we found highest stiffness in the first 0.2 μm to contact the probe and that greater indentation depths resulted in decreased overall stiffness. Note that mutant tissues are consistently softer that controls. p values of two-tailed t-tests comparing control and experimental measurements at 0.5 μm indentation depth were <0.01 in points 2, 3, 4, 5, 7 and 9, and <0.05 in points 6, 8 and 11. p values at 1 μm indentation depth were <0.01 in points 2, 3, 4, 5, 7, 9 and 11, and <0.05 in points 6 and 8. Image is a composite of several focal planes. (TIF) [file pgen.1005763.s008.tif]
