# Supplementary material for: ECM-Regulator timp Is Required for Stem Cell Niche Organization and Cyst Production in the Drosophila Ovary
Source: PLoS Genet. 2016 Jan 25;12(1):e1005763. doi: 10.1371/journal.pgen.1005763 (PMC4725958; doi:10.1371/journal.pgen.1005763)
Supplement: S2 Table — Differences in length-to-width ratios between control and mutants are statistically significant (p value of two-tailed t-tests <0.001 for 14-day old ovaries). Please refer to Fig 6 for examples of the different phenotypic classes. (DOCX) [file pgen.1005763.s010.docx]

**Table S2. Quantification of germarium shape, terminal filament position and stalk cell phenotypes in controls, *timp* mutants and *timp* mutants carrying a *UASt-timp* transgene**.

| Genotype | Days AE^a^ | % Abnormal  germ. shape^b^ (n) | Length-to-width ratio | % Abnormal  TF Position^c^ (n) | % Abnormal  Stalk length^d^ (n) |
| --- | --- | --- | --- | --- | --- |
| *timp^28^*/TM3 | 1-  7-  14- | 0 (35)  0 (31)  4.5 (22) | n. d.  2.27:1±0.33  1.94:1±0.35 | 0 (35)  0 (31)  0 (22) | 14.3 (35)  0 (27)  0 (19) |
| *timp^28^*/Df ED5472 | 1-  7-  14- | 0 (74)  6.1 (180)  59.1 (44) | n. d.  2.12:1±0.33  1.45:1±0.29 | 0 (78)  5.1 (178)  52.3 (44) | 50 (78)  42.6 (183)  27.3 (33) |
| *UASt-timp/+;*  *timp^28^*/Df ED5472 | 1-  7-  14- | 0 (29)  0 (45)  27 (37) | n. d.  n. d.  n. d. | 0 (29)  0 (45)  10.8 (37) | 33.3 (33)  9.5 (42)  7.9 (38) |

(n= sample size)

^a^ Samples were collected and processed 1-, 7- and 14-days after eclosion (AE) from the pupa.

^b^ Abnormal germ. shape refers to the percentage of germaria with a length-to-width ratio ≤1.5:1±0.0

^c^ Abnormal TF position refers to the percentage germaria where the Terminal Filament-Cap cells structure is not positioned at the anterior tip of the germarium as determined by the AP axis of the ovariole.

^d^ Abnormal stalk length refers to the percentage of germaria containing interfollicular stalks longer than 9 cells.

n. d.: Not determined.

Differences in length-to-width ratios between control and mutants are statistically significant (*p* value of two-tailed t-tests <0.001 for 14-day old ovaries).

Please refer to Figure 6 for examples of the different phenotypic classes.
