# Supplementary material for: ECM-Regulator timp Is Required for Stem Cell Niche Organization and Cyst Production in the Drosophila Ovary
Source: PLoS Genet. 2016 Jan 25;12(1):e1005763. doi: 10.1371/journal.pgen.1005763 (PMC4725958; doi:10.1371/journal.pgen.1005763)
Supplement: S3 Table — Differences in the #cysts/germarium between control and mutants are statistically significant (p values of two-tailed t-tests <0.001 for 2-, 10- and 21-day old germaria). (DOCX) [file pgen.1005763.s011.docx]

**Table S3. Quantification of the number of GSCs and cyst per germarium in controls and *timp* mutants**.

| Genotype | Days AE^a^ | #GSCs/ germarium (n) | #cysts/ germarium |
| --- | --- | --- | --- |
| *timp^28^*/TM3 | 2-  10-  21- | n. d. (24)  2.5±0.51 (20)  2.7±0.6 (19) | 9.1±1.9  9.4±1.8  9.5±2.2 |
| *timp^28^*/Df ED5472 | 2-  10-  21- | n. d. (22)  3.1±0.34 (16)  2.7±1.3 (29) | 10.8±3.7  6.2±2.4  5.8±3.5 |

(n= sample size)

^a^ Samples were collected and processed 2-, 10- and 21-days after eclosion (AE) from the pupa.

n. d.: Not determined.

Differences in the #cysts/germarium between control and mutants are statistically significant (*p* values of two-tailed t-tests <0.001 for 2-, 10- and 21-day old germaria).
