# Supplementary material for: ECM-Regulator timp Is Required for Stem Cell Niche Organization and Cyst Production in the Drosophila Ovary
Source: PLoS Genet. 2016 Jan 25;12(1):e1005763. doi: 10.1371/journal.pgen.1005763 (PMC4725958; doi:10.1371/journal.pgen.1005763)
Supplement: S4 Table — 1-week old adult females yeasted for 2 days were transferred to 37°C for 30 minutes to induce clone production. Ovaries were fixed, stained and prepared for observation 3 days later. (DOCX) [file pgen.1005763.s012.docx]

**Table S4. Quantification of cyst production in 1-week old controls and *timp* mutants**.

| Genotype | # germaria analysed | #GSCs/ germarium | Germaria containing GFP^+^ cysts | Labelled  germaria/  GSC x100 |
| --- | --- | --- | --- | --- |
| *hsFLP/+; UAS-mCD8::GFP/+; timp^28^ Act>y^+^>Gal4*/TM6B | 216 | 2.7±1.4 | 18.5% | 6.6 |
| *hsFLP/+; UAS-mCD8::GFP/+; timp^28^ Act>y^+^>Gal4*/Df ED5472 | 202 | 2.6±1.3 | 10.4% | 4 |

1-week old adult females yeasted for 2 days were transferred to 37^o^C for 30 minutes to induce clone production. Ovaries were fixed, stained and prepared for observation 3 days later.
