# Supplementary material for: ECM-Regulator timp Is Required for Stem Cell Niche Organization and Cyst Production in the Drosophila Ovary
Source: PLoS Genet. 2016 Jan 25;12(1):e1005763. doi: 10.1371/journal.pgen.1005763 (PMC4725958; doi:10.1371/journal.pgen.1005763)
Supplement: S1 Text — List of supporting figures and tables with additional experimental procedures for Fly Stocks, Preparation of fixed ovaries for observation/antibodies used, Preparation of live ovaries for observation, Transmission Electron Microscopy (TEM), Collection of Drosophila ovaries for proteome analysis, iTRAQ quantitation, LC-MS/MS Analysis, DIGE analysis, GO analysis of the differentially expressed proteins, Zymography assay and Atomic Force Microscopy analysis. (DOCX) [file pgen.1005763.s013.docx]

**SUPPORTING INFORMATION FOR:**

**Stem cell niche organisation and tissue homeostasis in the *Drosophila* ovary depends on *timp*-mediated ECM regulation.**

John R. Pearson, Federico Zurita, Laura Tomás-Gallardo, Alfonsa Díaz-Torres, María del C. Díaz de la Loza, Kristian Franze, María D. Martín-Bermudo and Acaimo González-Reyes*

*Correspondence to: AG-R ([agonrey@upo.es](mailto:agonrey@upo.es))

**Supporting figures and legends:**

**S1 Fig LTQ-Orbitrap analysis of wild-type ovaries confirms a shared proteome with *ovo^D1^* ovaries.**

(This supporting figure is related to Fig 1).

**S2 Fig. 2D-DIGE validation of identified candidates and gel zymography assay of collagenase activity.**

(This supporting figure is related to Fig 1 and Fig 2).

**S3 Fig. Collagen IV-FITC degradation is MMP-dependent.**

(This supporting figure is related to Fig 2).

**S4 Fig. The distribution of core ECM constituents in the ovarian basement membrane is not visibly affected in t*imp* mutant females.**

**S5 Fig. Quantification of core ECM constituents in the ovarian basement membrane.**

**S6 Fig. Transmission electron microscopy analysis of *timp* ovaries.**

**S7 Fig. FRAP analysis of Collagen IV:GFP.**

**S8 Fig. Determination of ovariole tissue stiffness at different indentation depths.**

(This supporting figure is related to Fig 5).

**Supporting tables:**

**S1 Table. Global list of quantitated proteins and identified genes in the iTRAQ, LTQ and 2D-DIGE analyses**.

(This supporting table is related to Fig 1, S1 Fig and S2 Fig).

**S2 Table. Quantification of germarium shape, terminal filament position and stalk cell phenotypes in control, *timp* mutant and *timp* mutants carrying a *UASt-timp* transgene**.

(This supporting table is related to Fig 5 and Fig 6).

**S3 Table. Quantification of the number of GSCs and cyst per germarium in controls and *timp* mutants.**

(This supporting table is related to Fig 5 and Fig 6).

**S4 Table. Quantification of cyst production in 1-week old controls and *timp* mutants**.

(This supporting table is related to Fig 5 and Fig 6).

### Supporting Experimental Procedures

**Fly stocks**

Flies were grown on standard media for *Drosophila*. The *timp* gene is nested in the *Synapsin (Syn)* locus*,* a genetic arrangement that is evolutionary conserved. Since a deletion or point mutation that only eliminates *timp* is not available, we generated a *timp* null condition by combining a ~15 kb deletion of that removes both *timp* and *synapsin* (*timp^28^*, a gift from A. Page-McCaw) with Df(3R)ED5472, which eliminates, among other genes, the *timp* locus (Bloomington *Drosophila* Stock Centre). Importantly, females bearing a deletion only affecting *synapsin* (*syn^27^*; [1]) over Df(3R)ED5472 display none of the phenotypes associated with the removal of *timp* (not shown). The presence of a UASt-*timp* and Heat-Shock (HS) Gal4 transgenes in *timp^28^*/Df(3R)ED5472 mutant females grown at 25ºC was sufficient to significantly restore normal ovary morphology but had no effect on other *timp* phenotypes such as wing blisters (Table S2 and data not shown). Surprisingly, a similar, albeit weaker effect was observed when UASt-*timp* was present in the absence of a GAL4 transgene, suggesting that leaky *timp* expression from the UAS-controlled insertion is sufficient to partially attenuate some of the phenotypes associated with the loss of the endogenous t*imp* gene

Other strains used are the following:

- For the FRAP and ECM analyses we have used GFP-tagged versions of the α2-Collagen IV chain, encoded by the *viking* gene (line G00205) and the Perlecan protein, encoded by the *terribly-reduced optic lobes* (*trol*) gene (line G00022; FlyTrap; <http://flytrap.med.yale.edu/>). Both protein traps target the endogenous loci and are homozygous viable, validating their use as reporters for gene activity.

- *UASt-timp* [2].

- *c587-Gal4*, a germarium-specific GAL4 line [3].

**Preparation of fixed ovaries for observation. Antibodies used.**

Antibody, DNA and rhodamine-phalloidin stainings were performed according to standard procedures. Detailed protocols are available upon request. DNA was counterstained with Hoechst (Sigma, 5 mg/ml). Antibodies were used at the following concentrations: mouse monoclonal anti-Fas3 (obtained from the Developmental Studies Hybridoma Bank, DSHB, University of Iowa) 1/20; rabbit polyclonal anti-Vasa (a gift from R. Lehmann), 1/3000; mouse monoclonal anti-Hts (DSHB), 1/25; mouse monoclonal anti-MMP1 hemopexin domain (DSHB clone 14A3D2), 1/10; mouse monoclonal anti-MMP1 catalytic domain antibodies (DSHB clones 3A6B4, 3B8D12 and 5H7B11) used as a 1:1:1 cocktail at a 1/4 final concentration; mouse monoclonal anti-Lamin C (DSHB), 1/40; Rabbit anti-Collagen IV, 1/5000 [4]; Rabbit Anti-laminin-beta and rabbit Anti-laminin-gamma, 1/500 [5]; Rabbit Anti-Perlecan (a gift from S. Baumgartner), 1/2000. FITC, Cy2, Cy3 and Cy5 conjugated secondary antibodies (Jackson Laboratories) were used at a final concentration of 1/100. Images were collected using Leica laser-scanning Confocal microscopes. Images were assembled using Adobe Photoshop and labeled in Adobe Illustrator.

**Preparation of live ovaries for observation.**

Individual ovarioles were dissected in Schneider medium supplemented with streptomycin and insulin as described previously [6]. Images were obtained on a Leica SP5 confocal microscope and processed utilizing the following software: IMARIS (Bitplane, Oxford, UK), ImageJ, Adobe Photoshop and Adobe Illustrator.

*Fluorescence Recovery After Photobleaching (FRAP)*

Pre-bleach images were collected using low laser power at 488 nm (typically below 15% laser power depending on sample signal intensity). Several regions of interest per ovariole were then bleached at 100% laser power for three 2 second scans (400 Hz, one line/frame average). After bleaching, images were collected from the treated regions and from adjacent, untouched areas at 20-minute intervals for 2 hours. Fluorescence intensity in the selected regions was quantitated. Recovery of fluorescence is expressed in arbitrary units over time. Because the ECM present in the cultured egg chambers keeps accumulating new material synthesized by the underlying follicular epithelium during the duration of the experiment, control regions show an evident increase in fluorescence intensity from stage 2-3 onwards**.**

**Transmission Electron Microscopy (TEM)**

**TEM** samples were prepared following standard procedures. Briefly, ovaries were dissected in Phosphate Buffer Saline + 0.1% Tween-20 and fixed for 2 hours at 4ºC in 3% glutaraldehyde/l% paraformaldehyde (vol./vol.) in 0.05 M cacodylate buffer (pH 7.4). After three 10 min. washes in cacodylate buffer 0.1 M at 4ºC, ovaries were postfixed for 1 hour at 4ºC in darkness in 1% OsO_4_, 1% K_3_Fe[CN]_6_ in water. Ovaries were then rinsed three times in distilled water at 4ºC and stained for 2 hours at room temperature (RT) in darkness in 0.5% uranyl acetate. After staining, ovaries were rinsed again in distilled water and dehydrated through an ethanol rising series (50%, 70%, 90% and 3x100%; 10 min. each) at RT. Ovaries were then infiltrated with Embed 812 resin (Electron Microscopy Sciences) as follows: Embed 812/ethanol 100%. 1:2, 1:1 and 2:1 for 1 hour at RT each, and in Embed 812 overnight at 4ºC. The resin-embedded specimens were polymerized by incubation in fresh Embed 812 during 48 hours at 60ºC in flat plastic embedding molds. The inclusion blocks were cut in 50-70 nm thick sections with a DIATOME diamond-blade fixed on a Reichert Jung Ultramicrotome and mounted on copper grids. Sections were counterstained with 1% uranyl acetate in 50% ethanol for 1 min. and then stained with lead citrate for 5 min. in a CO_2_-free atmosphere [7]. Sections were examined with a Zeiss EM902 electron microscope at 80Kv, and photographed at 50.000x magnification.

**Collection of *Drosophila* ovaries for proteome analysis**

0-3 day-old females were yeasted for 3 days prior to dissection. Ovaries were dissected in PBS and immediately frozen in liquid nitrogen.

The iTRAQ experiment was performed after collecting two biological replicas each of control and experimental flies. The number of ovary pairs utilized per replica is shown below:

| **iTRAQ Replica #** | **1** | **2** |
| --- | --- | --- |
| Control  *ovo^D1^*/*w^1118^*; *timp^28^*/TM3 | 235 | 312 |
| Experimental  *ovo ^D1^*/*w^1118^*; *timp^28^*/Df(3R)ED5472 | 123 | 162 |

For the LTQ-Orbitrap proteome analysis, we dissected two biological replicas, each containing 10 ovary pairs from *w^1118^* females. Ovaries were disrupted in 200 μL of Lysis buffer (7M Urea, 2M Thiourea, 4% CHAPS, 200 mM DTT) using the Sample Grinding kit (GE Healthcare). After disruption, samples were centrifuged for 20 minutes at 4ºC at 10,000 g.

For the 2D-DIGE analysis, we collected four biological replicas of control and experimental females. The following number of ovary pairs per replica was used:

| **2D-DIGE Replica #** | **1** | **2** | **3** | **4** |
| --- | --- | --- | --- | --- |
| Control  *w^1118^;; timp^28^*/TM3 | 25 | 30 | 20 | 29 |
| Experimental  *w^1118^;; timp^28^*/Df(3R) ED5472 | 67 | 74 | 36 | 72 |

Ovaries were disrupted in 200 μL of Lysis buffer (7M Urea, 2M Thiourea, 4% CHAPS, 200 mM DTT) using the Sample Grinding kit (GE Healthcare). After disruption, samples were centrifuged for 20 minutes at 4ºC at 10,000 g.

**iTRAQ quantitation (I): Sample Preparation, Protein Digestion, tagging with iTRAQ-4-plex®, Peptide fractionation and RP-LC-MALDI TOF/TOF MS**

In order to filter out differences caused by experimental variation, protein quantitation was carried using four independently collected samples from control and experimental ovaries [8]. Overall protein concentration was estimated using the Micro BCATM protein assay kit (Pierce, Thermo Scientific) with bovine serum albumin as a standard. An overview of the experimental design is shown in Figure 1. For digestion, 50 µg of protein from each condition was precipitated following the methanol/chloroform method. Protein pellets were resuspended and denatured in 20 µl 50mM triethylammonium bicarbonate (TEAB)/50% trifluoroethanol (TFE), (Sigma-Aldrich), reduced with 10mM Tris(2-carboxyethyl) phosphine (TCEP, AB SCIEX), pH 8.0, at 60°C for 60 min, followed by incubation with 2 µL cysteine-blocking reagent (methyl methanethiosulfonate; MMTS, Pierce) for 10 min at room temperature. Samples were diluted up to 200 µL with 50mM TEAB, to decrease TFE concentration. Digestions were initiated by adding 5 µL (1 µg/µL) sequencing grade-modified trypsin (Sigma-Aldrich) to each sample in a 1/10 ratio (w/w), and they were subsequently incubated overnight at 37°C on a shaker. Sample digestions were evaporated to dryness.

Each peptide solution was labeled for 2 hours at room temperature with half a unit of iTRAQ Reagent Multiplex kit (AB SCIEX, Foster City, CA, USA) previously reconstituted in 70 μl of ethanol. iTRAQ labeling was performed using a 4-plex design with the two biological replicates for each condition (control *vs* experimental samples). In the labeling reaction, tags 116 and 117 were used for control conditions and 114 and 115 for experimental replicates. Samples with the same protein content, labeled respectively with 114, 115, 116 and 117 iTRAQ reagents, were subsequently combined and the labeling reaction stopped by evaporation in a Speed Vac.

The digested, labeled and pooled samples were studied in detail by RP-LC-MALDI TOF/TOF MS. In the first place, peptide separation was performed at basic pH on a Fortis C18 column, 100 mm x 2.1 mm, 5 μm, (Fortis Technologies, Germany). About 200 μg of digested peptides from each labeling reaction were manually injected onto the column at 150 μL/min. with eluent A (10 mM aqueous ammonium hydroxide, pH 10 adjusted with formic acid). Peptide elution was accomplished by HPLC (Knauer GmbH, Berlin, Germany) with a gradient of 0-55 % B during 55 min. Eluent B consisted of 10 mM aqueous ammonium hydroxide in 80% acetonitrile, pH 10. Detection of peptide elution was performed at 214 nm. Fractions of approx. 150 μL were collected every minute and subsequently pooled into 6 fractions according to chromatogram complexity. Finally, combined fractions were evaporated to dryness in a vacuum concentrator (Model 5301, Eppendorf AG, Hamburg, Germany).

The selected fractions were reconstituted with 0.1% heptafluorobutyric acid (HFBA) and 16 μL aliquots of each selected fraction (~3 µg) were injected with a TEMPO nanoMDLC HPLC (AB SCIEX, Foster City, CA, USA) onto a ProteCol trap column (10 mm x 300 µm i.d.) (SGE Analytical Science, Australia) for peptide pre-concentration. Peptides were then washed for 4 min. with 0.1% aqueous heptafluorobutyric acid at 15 μL/min. and eluted onto the Onyx monolith C18 column (150 mm x 0.1 mm i.d.) (Phenomenex, Aschaffenburg, Germany) at a flow rate of 1 μl/min. Mobile phase composition was (A) 0.1% TFA in H2O and (B) 0.1% TFA in 80% ACN. The gradient employed was a 60 min. linear gradient from 0 to 25% B followed by a 10 min. isocratic elution at 100% B.

The eluting peptides were mixed post-column with a α-cyano-4-hydro-cinnamic acid MALDI matrix at a ratio of 1:1.5 (v:v). The resulting flow rate for the spotting procedure was 1.5 μL/min. The matrix concentration used was 3 mg/ml in 70% acetonitrile and 0.1% TFA aqueous solution. MALDI-plate spotting was performed by a Suncollect (SunChrom, Germany GmbH) fraction collector. Spots were deposited every 6 seconds on the MALDI-plate (Opti-TOF™ LC MALDI Insert, AB SCIEX, Foster City, CA, USA).

**iTRAQ quantitation (II): Mass spectrometry analysis**

A MALDI TOF/TOF 4800 (AB SCIEX, Foster City, CA, USA) mass spectrometer was used for acquisition and data processing. MS spot data was acquired in positive reflector ion mode in the mass range of 800-3500 m/z by accumulation of 1200 laser shots. In addition to the default calibration, MS spectra were internally calibrated using Angiotensin-II (Sigma) that was added to the matrix to a concentration of 10 fmol/μL. The list of precursors for MS/MS analysis was automatically generated by the instrument software according to the following selection criteria: minimum signal to noise ratio 80; precursor mass tolerance between spots ± 200 ppm; minimum chromatogram peak width of two spots and a maximal of 8 precursors per spot. An exclusion filter was used to eliminate the internal mass standard. MS/MS spectra were generated by 2 kV collisions with air. Maximal 2000 laser shots were accumulated for a MS/MS spectrum. Stop conditions for MS/MS were defined as a minimal number of 10 peaks above 50 S/N with at least 25 accumulated sub-spectra.

Raw files containing a peak list of the precursors and fragment ions were filtered and exported with the ABI-Extractor tool (Peaks-Bioinformatics Solutions, Canada). Protein identification and quantitation were done utilizing the MASCOT v2.3.01 (Matrix Science, London, UK) and the PHENYX v2.6 (GeneBio, Geneva, Switzerland) search engines. The search was performed against the *Drosophila* *melanogaster* database of UniProtKB/Swiss-Prot (42,034 sequences, 24,968,334 residues). A concatenated target-decoy database search strategy was used to estimate the false positive rate below 1%, which boosted the reliability of the data. The following search parameters were used: enzyme, trypsin; allowed missed cleavages, 1; fixed modifications, iTRAQ4plex (N-term and K); variable modifications, beta-methylthiolation of cysteine, oxidation of methionine and acetylation (N-term); mass tolerance for precursors was set to ± 100 ppm and for MS/MS fragment ions to ± 0.3 Da. The confidence interval for protein identification was set to ≥ 95% (p<0.05) and only peptides with an individual ion score above the 1% FDR threshold were considered correctly identified. Only proteins having at least three quantitated peptides were considered in the final quantitation.

The ratios of relative quantities control/experimental for the Mascot search engine were calculated for each protein entry and the geometric mean used to classify them as differentially abundant or not. The total average ratio was calculated as the geometric mean of at least two values from the search engine. 48 proteins with significantly different levels in control *vs.* *timp* mutant ovaries were identified (i. e., with control/experimental average expression ratios ≤0.50 and ≥1.50; Table S1).

**LC-MS/MS Analysis of wild-type ovaries on a LTQ-Orbitrap: Sample preparation, protein digestion and mass spectrometry analysis**

Protein samples were reduced with 10mM dithiothreitol (DTT) in 25 mM ammonium bicarbonate for 30 min at 56ºC and subsequently alkylated with 55 mM iodoacetamide in 25mM ammonium bicarbonate for 60 min in darkness. Samples were then digested with 1:20 sequencing grade trypsin (Roche Molecular Biochemicals) in 25 mM ammonium bicarbonate (pH 8.5) overnight at 37ºC. After digestion, 150 µg of sample were resuspended in 20 µl 2% formic acid. 3 µg of the peptide mixtures were separated on-line with an Easy-nLC (Proxeon) using a 160 x 75 µm NS-AC-11-dp3 C18 column (BioSphere) and a Pre-Column Easy-column C18-A1 (Proxeon) at a flow rate of 250 nL/min with a 245 min gradient: 190 min 2-30% of B phase, 40 min 30-40% of B phase, 10 min 40-90% of B phase and 4 min 99% of B phase. The composition of solvent A was: formic acid 0.1%, acetonitrile 2%, water 98% and solvent B was 99.9% acetonitrile with 0.1% formic acid. An electrospray voltage of 1.7 kV versus the inlet of the mass spectrometer was used.

Peptides were analyzed using a linear trap Orbitrap Velos (LTQ Orbitrap Velos) hybrid mass spectrometer (Thermo Electron Corp., Bremen, Germany). Ion transmission into the linear trap and further to the Orbitrap Velos was automatically optimized for maximum ion signal for m/z 400-1200 using Automatic Gain Control (AGC). The AGC target settings were: for Ion Trap (IT) Full Ms and Ms^n^ 3 x 10^4^ and 1 x 10^4^, respectively; and for Fourier Transform (FT) 1 x 10^6^ and 5 x 10^4^. The scan time settings for IT Full Ms and Ms^n^ were 10 and 100 ms, respectively; and for FT Full Ms and Ms^n^ were 500 and 1000 ms. The resolving power of the FT mass analyzer was set at 60000.

The mass spectrometer was operated in the data-dependent mode to automatically switch between full MS and MS/MS acquisition. The parameters for ion scanning were Full-scan MS (400-1200 m/z) plus top 10 peaks MS2. In data-dependent LC/MS2 experiments dynamic exclusion was used with one repeat count, 30 s repeat duration, and 30 s exclusion duration. For MS/MS, precursor ions were activated using 38% normalized collision energy at the default activation q of 0.25.

Proteome Dicoverer software (Thermo, version 1.2.0.208) was used to search against the *Drosophila* *melanogaster* database of UniProtKB/Swiss-Prot (42,034 sequences, 24,968,334 residues) using Mascot (version 2.3.0). Search parameters were the following: precursor mass tolerance, 10 ppm; fragment mass tolerance, 0.8 Da; missed cleavage sites, 1; static modification, carbamidomethyl cysteine; and dynamic modifications, methionine oxidation. Acceptance criteria: High peptide confidence (p<0.05) and FDR< 0.1.

**DIGE analysis: Sample preparation, fluorophore labeling, 2D electrophoresis and protein identification**

Prior to 2D electrophoresis, samples were treated with the 2-D Clean-Up kit (GE Healthcare) and resuspended in Cell lysis solution (7M urea, 2M thiourea, 4% CHAPS, 20 mM tris-HCl pH 9.5). Protein concentrations were determined using the RC DC Protein assay kit (Bio-Rad) with Bovine Serum Albumina as standard. A total of 50 μg of proteins from each condition were labeled with 400 pmol of Cy3 or Cy5 dyes following the manufacturer’s instructions. As internal standards, 25 μg of each sample were mixed and labeled with the Cy2 dye. For Isoelectro focusing (IEF) an Ettan IPGphor I System was used. 24-cm, pH range 3-10, Immobiline DryStrip gels were rehydrated for 11 hours in rehydration buffer (7M urea, 2M thiourea, 4% CHAPS, 0.5% IPG buffer pH 3-10, 20mM DTT) containing pooled protein samples. IEF was performed as follows: 1 h-500V step-n-hold, 1 h-1000V gradient, 3 h-8000V gradient and 4 h-8000V step-n-hold until 46.000 Vhr were reached. After completion of the IEF, cysteine sulfhydryl groups were reduced and carbamidomethylated by strip incubation for 15 min. in equilibration solution I (50 mM Tris pH 8.8, 6 M urea, 30% glycerol, 2% SDS, 10 mg/mL DTT) and 15 min. in equilibration solution II (solution I plus 25 mg/mL iodoacetamide instead of DTT). The second dimension was performed on 12% SDS-PAGE gels using the Ettan Dalt Six electrophoresis system. Fluorescent gel images were scanned with a Typhoon 9400 Scanner and analyzed with the DeCyder Differential Analysis software 7.0. Unless otherwise stated, equipment, software and chemicals were provided by GE Healthcare. Gel images were processed with 10,000 expected spots. Spots with volumes smaller than 30,000 and slopes larger than 1.5, mostly dust artifacts, were excluded from further analysis. Changed protein spots with p-values ≤0.05 were picked for finger-printing identification. Silver staining was carried out as described with minor modifications [9].

Protein spots were excised from gels manually and transferred to pierced V-bottom 96-well polypropylene microplates (Bruker Daltonik, Bremen, Germany) loaded with ultrapure water. Samples were digested automatically using a Proteineer DP robot ran by the dpControl 1.2 software (Bruker Daltonik) according to a established protocol [10] with minor variations: gel plugs were submitted to reduction with 10 mM dithiothreitol (GE Healthcare) in 50 mM ammonium bicarbonate (Sigma Chemical) and alkylation with 55 mM iodoacetamide (Sigma Chemical) in 50 mM ammonium bicarbonate. Gel pieces were then rinsed with 50 mM ammonium bicarbonate and acetonitrile (Merck) and dried under a stream of nitrogen. Modified porcine trypsin (Promega) at a final concentration of 8 ng/µl in 50 mM ammonium bicarbonate was added to digest the dried gel pieces at 37 ºC for 8 h. Finally, 0.5% trifluoroacetic acid (Sigma Chemical) was added for peptide extraction, and the resulting digestion solutions transferred by centrifugation to V-bottom 96-well polypropylene microplates (Greiner Bio-One).

MALDI samples were prepared by mixing equal volumes of the above digestion solutions and a matrix solution composed of α-cyano-4-hydroxycinnamic acid (Bruker Daltonik) in 50% aqueous acetonitrile and 0.25% trifluoroacetic acid. This mixture was deposited onto a 600 µm AnchorChip pre-structured MALDI probe (Bruker Daltonik) and allowed to dry at room temperature. Samples were automatically analyzed in an Ultraflex MALDI-TOF/TOF mass spectrometer (Bruker Daltonik) with an automated analysis loop using internal mass calibration, under the control of flexControl 2.2 software (Bruker Daltonik). In a first step, the MALDI-MS spectra were acquired by averaging 300 individual spectra in the positive ion reflector mode at 50 Hz laser frequency with a mass range of 800 to 4000 Da. Internal calibration of MALDI-MS mass spectra was performed using two trypsin autolysis ions with m/z= 842.510 and m/z= 2211.105. In a second step, precursor ions exceeding a threshold signal-to-noise ratio in the MALDI-MS mass spectrum were subject to fragment ion analysis in the tandem (MS/MS) mode. Precursors were accelerated to 8 kV and selected in a timed ion gate. 19 kV in the LIFT cell further accelerated fragment ions generated by laser-induced decomposition of the precursor and their masses were analyzed after passing the ion reflector to average 1000 spectra. For MALDI-MS/MS, calibrations were performed with fragment ion spectra obtained for the proton adducts of a peptide mixture covering the 800-3200 m/z region. Automated analysis of mass data was performed using the flexAnalysis 2.2 software (Bruker Daltonik). MALDI-MS and MS/MS spectra were manually inspected in detail and reacquired, recalibrated and/or re-labeled when necessary using the above programs as well as home-made software.

MALDI-MS and MS/MS data were combined in the BioTools 3.0 program (Bruker Daltonik) to search non-redundant protein databases (NCBInr, ~10^7^ entries, National Center for Biotechnology Information, Bethesda, US; or SwissProt, ~5 x 10^5^ entries, Swiss Institute for Bioinformatics, Switzerland) using the Mascot software (Matrix Science, London, UK) [11]. Other relevant search parameters were set as follows: enzyme, trypsin; fixed modifications, carbamidomethyl; allow up to one missed cleavages; peptide tolerance ±20 ppm; MS/MS tolerance ±0.5 Da.

**GO analysis of the differentially expressed proteins**

In order to perform ontological and functional studies of the genes coding for differentially-expressed proteins, we generated a list of candidate genes that was evaluated using PANTHER (http://www.pantherdb.org/) and GeneCodis (version 3; http://genecodis.cnb.csic.es/) tools. The PANTHER system [12] allows the classification of gene products according to gene families, gene ontologies or protein networks and pathways. GeneCodis [13-15] determines biological annotations or combinations of annotations with a statistically-significant enrichment within a gene list. The GeneCodis analysis applied a hypergeometric test followed by permutation-based correction as a statistical test to determine which annotations are significantly enriched in an input Differentially Expressed Gene list with respect to a reference list. In our case, we chose a list of *Drosophila melanogaster* whole transcriptomes available from public databases.

**Zymography assay**

0-3 day-old females were yeasted for 1-2 days prior to dissection. Ovaries were dissected in Schneider’s medium supplemented with Fetal Bovine Serum (15% vol/vol) containing streptomycin/penicillin (10,000 U ml^-1^ of penicillin G–sodium, 10,000 μg ml^−1^streptomycin sulfate in 0.85% saline) to a final concentration of 0.6x [6]. Ovaries were incubated for 2 hours in 200 µl of the above culturing cocktail in the presence of 10 µl of Collagen IV-FITC (Collagen, type IV from human placenta, fluorescein conjugate; Invitrogen). Ovaries were then fixed in 4% paraformaldehyde for 20 minutes and stained with Rhodamine-Phalloidin to visualize filamentous actin. According to the manufacturer’s information, the FITC fluorescence on intact Collagen IV substrate is quenched due to the high density of conjugated FITC molecules, In contrast, enzyme-dependent Collagen IV degradation results in the separation of dye molecules thus allowing detectable FITC emissions. In a parallel experiment and prior to the addition of Collagen IV-FITC, control ovaries were pre-incubated for 30’ to avoid cytotoxicity with 1mM 1, 10-Phenanthroline (Invitrogene), an MMP inhibitor.

In order to quantify the fluorescence of control and experimental samples, image series were captured using identical settings. Color depth was set to 12-bit and configured to avoid saturated pixels as much as possible. Depth (z) thresholds were set well above and below each ovariole to guarantee that the complete tissue was captured. Sections were taken at 0.63 µm intervals (optimal). FITC signal was captured using the default Leica FITC configuration and low laser intensity. After recording position and z-depth ranges, image series were captured automatically for each slide. Analysis was only performed for those ovarioles imaged outside of the muscle sheath that normally surrounds them. All image stacks were pre-processed using the standard background subtraction function of ImageJ (default settings; 50 pixel radius). Measurements were taken using IMARIS software with the "Measurement points” tool. Ovarioles were subdivided into different sections (terminal filament, germarial region 1-2a, germarial region 2b-3, 1^st^ stalk, 1^st^ egg chamber, 2^nd^ stalk, 2^nd^ egg chamber, etc.) and each section was measured at several points along the anterior-posterior axis. Three measurement series were taken for each ovariole. Depth was selected automatically by IMARIS based on FITC channel intensity.

**Atomic Force Microscopy analysis**

We measured the stiffness of *ex-vivo* ovarian tissues immobilized in Petri dishes containing *Drosophila* culture media. Muscle sheath was removed from the dissected ovarioles to make sure that the AFM cantilever was in direct contact with the basement membrane that surrounds the tissues, which were tested by AFM within 30 minutes of dissection. Monodisperse polystyrene beads (diameter 5.46 ± 0.12 µm, microParticles GmbH, Berlin, Germany) were glued to silicon cantilevers with a nominal spring constant of 0.1 N/m (PPP-BSI, Nanosensors, Neuchatel, Switzerland). Cantilevers were mounted on a JPK Nanowizard II AFM (JPK Instruments AG, Berlin, Germany), which was set up on an inverted optical microscope (Nikon Eclipse TE2000-U, Nikon, Japan). Exact cantilever spring constants were determined using the thermal noise method included in the AFM software. Freshly dissected samples were measured under visual control [17]. Force-distance-curves were taken at an approach speed of 10 µm/s and a maximum force *F* = 6 nN. Force–distance curves were analyzed for an indentation depth δ = 0.2, 0.5 and 1.0 µm using a custom algorithm based in Matlab [18] (MathWorks, Natick, USA), which fits the Hertz model to the data: , where the apparent elastic modulus is a measure of stiffness, *R* is the radius of the indenter, *E* is the Young’s modulus, and ν is the Poisson’s ratio. Normal distribution of AFM measurements was confirmed using the Kolmogorov-Smirnov test. The statistical significance of the differences between experimental and control values was evaluated using two-tailed t-tests.


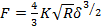

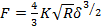

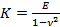

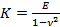


### References

1. Godenschwege TA, Pohar N, Buchner S, Buchner E. Inflated wings, tissue autolysis and early death in tissue inhibitor of metalloproteinases mutants of Drosophila. Eur J Cell Biol. 2000;79(7):495-501. Epub 2000/08/29. PubMed PMID: 10961449.

2. Page-McCaw A, Serano J, Sante JM, Rubin GM. Drosophila matrix metalloproteinases are required for tissue remodeling, but not embryonic development. Dev Cell. 2003;4(1):95-106. Epub 2003/01/18. doi: S1534580702004008 [pii]. PubMed PMID: 12530966.

3. Kai T, Spradling A. An empty Drosophila stem cell niche reactivates the proliferation of ectopic cells. PNAS. 2003;100:4633-8.

4. Lunstrum G, Baechinger H-P, Fessler LI, Duncan K, Nelson R, Fessler J. Drosophila basement membrane procollagen IV. I. Protein characterization and distribution. J BiolChem. 1988;263:18318-27.

5. Kumagai C, Kadowaki T, Kitagawa Y. Disulfide-bonding between *Drosophila* laminin β and γ chains is essential for α chain to form αβγ trimer. FEBS Lett. 1997;412(1):211-6. PubMed PMID: 9257722.

6. Prasad M, Jang AC, Starz-Gaiano M, Melani M, Montell DJ. A protocol for culturing Drosophila melanogaster stage 9 egg chambers for live imaging. Nat Protoc. 2007;2(10):2467-73. Epub 2007/10/20. doi: nprot.2007.363 [pii]

10.1038/nprot.2007.363. PubMed PMID: 17947988.

7. Reynolds ES. The use of lead citrate at high pH as an electron-opaque stain in electron microscopy. J Cell Biol. 1963;17:208-12. Epub 1963/04/01. PubMed PMID: 13986422; PubMed Central PMCID: PMC2106263.

8. Gan CS, Chong PK, Pham TK, Wright PC. Technical, experimental, and biological variations in isobaric tags for relative and absolute quantitation (iTRAQ). J Proteome Res. 2007;6(2):821-7. Epub 2007/02/03. doi: 10.1021/pr060474i. PubMed PMID: 17269738.

9. Yan JX, Wait R, Berkelman T, Harry RA, Westbrook JA, Wheeler CH, et al. A modified silver staining protocol for visualization of proteins compatible with matrix-assisted laser desorption/ionization and electrospray ionization-mass spectrometry. Electrophoresis. 2000;21(17):3666-72. Epub 2001/03/29. doi: 10.1002/1522-2683(200011)21:17<3666::AID-ELPS3666>3.0.CO;2-6 [pii]

10.1002/1522-2683(200011)21:17<3666::AID-ELPS3666>3.0.CO;2-6. PubMed PMID: 11271485.

10. Shevchenko A, Tomas H, Havlis J, Olsen JV, Mann M. In-gel digestion for mass spectrometric characterization of proteins and proteomes. Nat Protoc. 2006;1(6):2856-60. Epub 2007/04/05. doi: 10.1038/nprot.2006.468. PubMed PMID: 17406544.

11. Perkins DN, Pappin DJ, Creasy DM, Cottrell JS. Probability-based protein identification by searching sequence databases using mass spectrometry data. Electrophoresis. 1999;20(18):3551-67. Epub 1999/12/28. doi: 10.1002/(SICI)1522-2683(19991201)20:18<3551::AID-ELPS3551>3.0.CO;2-2. PubMed PMID: 10612281.

12. Thomas PD, Kejariwal A, Campbell MJ, Mi H, Diemer K, Guo N, et al. PANTHER: a browsable database of gene products organized by biological function, using curated protein family and subfamily classification. Nucleic Acids Res. 2003;31(1):334-41. Epub 2003/01/10. PubMed PMID: 12520017.

13. Carmona-Saez P, Chagoyen M, Tirado F, Carazo JM, Pascual-Montano A. GENECODIS: a web-based tool for finding significant concurrent annotations in gene lists. Genome Biol. 2007;8(1):R3. Epub 2007/01/06. doi: gb-2007-8-1-r3 [pii]

10.1186/gb-2007-8-1-r3. PubMed PMID: 17204154.

14. Nogales-Cadenas R, Carmona-Saez P, Vazquez M, Vicente C, Yang X, Tirado F, et al. GeneCodis: interpreting gene lists through enrichment analysis and integration of diverse biological information. Nucleic Acids Res. 2009;37(Web Server issue):W317-22. Epub 2009/05/26. doi: gkp416 [pii]

10.1093/nar/gkp416. PubMed PMID: 19465387.

15. Tabas-Madrid D, Nogales-Cadenas R, Pascual-Montano A. GeneCodis3: a non-redundant and modular enrichment analysis tool for functional genomics. Nucleic Acids Res. 40(Web Server issue):W478-83. Epub 2012/05/11. doi: gks402 [pii]

10.1093/nar/gks402. PubMed PMID: 22573175.

16. Herron GS, Werb Z, Dwyer K, Banda MJ. Secretion of metalloproteinases by stimulated capillary endothelial cells. I. Production of procollagenase and prostromelysin exceeds expression of proteolytic activity. J Biol Chem. 1986;261(6):2810-3. Epub 1986/02/25. PubMed PMID: 3005265.

17. Franze K, Francke M, Günter K, Christ AF, Körber N, Reichenbach A, et al. Spatial mapping of the mechanical properties of the living retina using scanning force microscopy. Soft Matter. 2011;7(7):3147. doi: 10.1039/c0sm01017k.

18. Christ AF, Franze K, Gautier H, Moshayedi P, Fawcett J, Franklin RJ, et al. Mechanical difference between white and gray matter in the rat cerebellum measured by scanning force microscopy. Journal of biomechanics. 2010;43(15):2986-92. Epub 2010/07/27. doi: 10.1016/j.jbiomech.2010.07.002. PubMed PMID: 20656292.
